# Self-Cleaning Bending Sensors Based on Semitransparent ZnO Nanostructured Films

**DOI:** 10.1021/acsaenm.3c00082

**Published:** 2023-05-03

**Authors:** Giuseppe Arrabito, Antonio Delisi, Giuliana Giuliano, Giuseppe Prestopino, Pier Gianni Medaglia, Vittorio Ferrara, Federica Arcidiacono, Michelangelo Scopelliti, Delia Francesca Chillura Martino, Bruno Pignataro

**Affiliations:** † Department of Physics and Chemistry—Emilio Segrè, University of Palermo, Viale delle Scienze 17, 90128 Palermo, Italy; ‡ Department of Industrial Engineering, University of Rome “Tor Vergata”, Via del Politecnico 1, 00133 Rome, Italy; § Department of Biological, Chemical and Pharmaceutical Sciences and Technologies (STeBiCeF), University of Palermo, Viale delle Scienze 16, 90128 Palermo, Italy; ∥ National Interuniversity Consortium of Materials Science and Technology (INSTM), UdR of Palermo, 50121 Florence, Italy

**Keywords:** ZnO nanostructures, piezotronics, photocatalysis, flexible sensors, wearable device

## Abstract

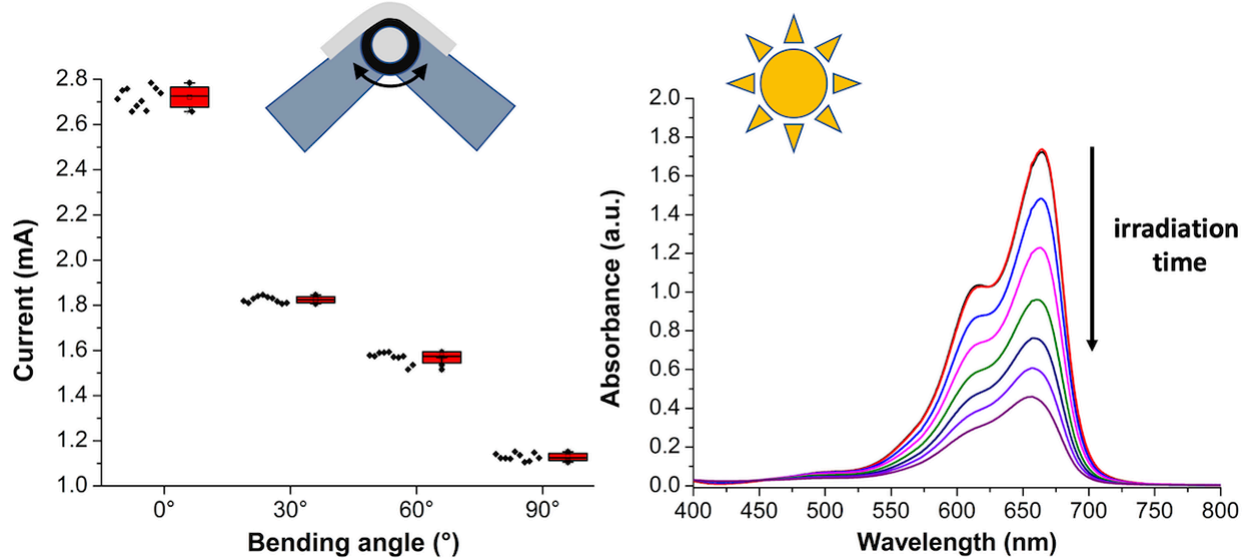

The design of multifunctional nanostructured materials is the key to the development of smart wearable devices. For instance, nanostructures endowed with both piezoelectric and photocatalytic activities could well be the workhorse for solar-light-driven self-cleaning wearable sensors. In this work, a simple strategy for the assembly of a flexible, semitransparent piezophotocatalytic system is demonstrated by leveraging rational wet chemistry synthesis of ZnO-based nanosheets/nanoflowers (NSs/NFs) under basic pH conditions onto flexible ITO/PET supports. A KMnO_4_ pretreatment before the ZnO synthesis (seeded ZnO) allows for the control of the density, size, and orientation of the NSs/NFs systems compared to the systems produced in the absence of seeding (seedless ZnO). The electrical response of the sensors is extracted at a 1 V bias as a function of bending in the interval between 0 and 90°, being the responsivity toward bending significantly enhanced by the KMnO_4_ treatment effect. The photocatalytic activity of the sensors is analyzed in aqueous solution (methylene blue, 25 μM) by a solar simulator, resulting in similar values between seedless and seeded ZnO. Upon bending the sensor, the photocatalytic activity of seedless ZnO is almost unaffected, whereas that of seeded ZnO is improved by about 25%. The sensor’s reusability and repeatability are tested in up to three different cycles. These results open up the way toward the seamless integration of bending sensitivity and photocatalysis into a single device.

## 1 Introduction

The development of wearable sensing technologies in the past two decades has resulted in integrated systems performing multiple analyses into a single device, aiming at comprehensive monitoring of the human physiological status.^1^ Materials for wearable sensors are becoming more and more sustainable, adaptive to the environment, thinner, and eco-friendly.^2^ Among them, piezoelectric and triboelectric systems constitute a well-established platform for flexible and wearable electronic devices.^3^ In particular, piezoelectric materials have been used for detection sensors in the context of biomechanical signal detection, for instance, body movements, heart rate, and breath.^4^ Importantly, one has to consider that most of the conventional inorganic piezoelectric materials (BaTiO_3_, PZT, and ZnO), albeit possessing a high piezoelectric constant, cannot withstand large deformation.^5^ For such reasons, they need to be combined with flexible organic substrates or used to fabricate composites with suitable polymers such as cellulose^6^ or polyvinylidene fluoride-trifluoroethylene.^7^ As an alternative, ITO/PET has been shown as a highly sensitive semitransparent pressure and strain sensor,^8^ usable for detecting finger bending movement by a purely piezoresistive mechanism based on the formation of cracks within the grains of the ITO material. By applying strain, the cracks are separated, leading to an increase in electrical resistance, whereas the backward movement permits the ITO to return to its initial resistance value.

A big issue that needs to be solved is the possible contamination of the sensor surfaces by potentially pathogenic systems of different natures, e.g., micropollutants, bacteria, and viruses. This could be a crucial factor not only for the functionality of the device itself but also for the safety of the end user/patient, as a contaminated device could even be a potential source of transmission, especially for viruses.^9^ Accordingly, the development of wearable sensor device technology would greatly benefit from engineering surfaces that can couple both the desired sensing functionality and nature-inspired self-cleaning features.^10^ Indeed, self-cleaning surfaces are able to remove surface-attached contaminants by a variety of mechanisms, relying on their superhydrophobic, superhydrophilic, or photocatalytic properties. To this aim, ZnO can well modulate the wettability of the sensor surface and is also featured with good photocatalytic properties,^11,12^ which could be beneficial for their photocatalytic properties, similar to semitransparent self-cleaning TiO_2_ coatings.^13^ A great boost in this technology is pursued by the emerging field of piezocatalysis^14^ that leverages piezoelectricity for the efficient conversion of mechanical energy derived from wind or wave motion or waste mechanical vibrations into useful electricity employed for electrochemical reactions, involving, for example, reactive oxygen species (ROS) suitable for water decontamination.^15^

To this aim, it would be essential to design a dual-use material, which could combine both sensing and self-cleaning activities by leveraging sunlight, which is an inexpensive source of energy.^16^ In this regard, research efforts have shown promising results through the development of piezocatalytic materials. For example, the MoS_2_ nanoflower-embedded self-poled PVDF nanocomposite film shows a power density higher than 40 mW/cm^3^ under human finger tapping^17^ in addition to optimal photodegradation capabilities of model colored contaminants (acridine orange, eosin Y, ethidium bromide, and rhodamine). Another example was shown by Liu et al., who added titania nanoparticles into tribolectric nanogenerators based on sponged polydimethylsiloxane (PDMS) films.^18^ In doing so, they observed that 0.05% titania permitted us to recover almost 90% of the device performance. Other studies investigated the CdS/ZnO nano-heterojunction for efficient dye wastewater decomposition,^19^ WS_2_ monolayers for piezophototronic methylene blue (MB) degradation,^20^ and eco-friendly BaTiO_3_ two-dimensional (2D) nanosheets for rhodamine B degradation.^21^

Among the piezophotocatalytic materials suitable for self-cleaning wearable sensors, ZnO nanostructures (*n-*ZnO) might constitute an ideal choice due to their immense versatility in piezotronic devices,^22^ along with excellent biodegradability^23^ and biocompatibility as reported by the Food and Drug Administration,^24^ which ultimately make them attractive also for wearable applications. ZnO can be synthesized following vapor or wet-chemical routes.^25^ The *n-*ZnO-based devices mostly find applications as nanogenerators, i.e., energy harvesters from the environment or sensors. Importantly, their properties are highly dependent on the nanostructure geometry, e.g., one-dimensional (1D) nanowires (NWs), tetrapods,^26^ nanoflowers (NFs),^27^ or 2D nanosheets (NSs),^28^ with the 2D structures featuring the highest pressure sensitivity. The piezoelectric properties of ZnO have also been investigated in the field of piezocatalytic applications, especially for organic pollutant degradation. However, the piezocatalytic activity of pure ZnO is not ideal for these applications due to its high band gap (3.37 eV), which limits its absorption in the visible spectrum and, as such, it has been improved through morphology modification,^29^ (e.g., NFs showing more efficiency vs. NWs^30^), oxygen vacancy introduction, doping,^31^ and semiconductor heterojunction coupling.^32^ In all of the above-mentioned reports, the photocatalytic activity of *n-*ZnO is improved by ultrasonication or strain application. However, to the best of our knowledge, the integration of *n-*ZnO demonstrating an ideal dual feature, i.e., both piezoelectric sensing and photocatalytic activity, into a single device has not yet been reported.

Herein, we leverage a rational, mild, and efficient wet chemistry procedure to realize reconfigurable *n-*ZnO on ITO for bending sensors coupling a piezoelectrical response with photocatalytic properties. First, a rational wet chemistry synthetic procedure is selected at a basic pH value, allowing for the formation of photocatalytic nanocubes (NCs) made of indium, tin, and zinc mixed oxides at the ITO surface. The employment of a KMnO_4_ seed layer before *n-ZnO* synthesis allows us to select between low-density ZnO NFs (seedless ZnO) and high-density 2D ZnO NSs (seeded ZnO) grown on the NCs. The two resulting engineered surfaces are characterized by good sensitivity toward bending as a result of their piezoelectric properties. The photocatalytic activity of the samples was demonstrated to be enhanced by bending in the case of seeded ZnO, along with good reusability after washing with ultrapure water for up to three cycles.

## 2 Experimental Section

### 2.1 ZnO Nanostructures Synthesis

Nanostructured ZnO films were grown onto precut (50 mm × 15 mm) ITO/PET commercial supports (Sigma-Aldrich, surface resistivity 60 Ω/sq). Before deposition, the ITO/PET supports were patterned by removing ITO on the lateral zone with 5 M HCl and then cleaned with ethanol and water for 15 min by an ultrasonic bath, leaving an ITO active area of 50 mm × 5 mm. The ZnO growth was carried out with a nutrient solution prepared in ultrapure deionized (DI) water with resistivity at 25 °C > 18.2 MΩ·cm (Smart2Pure Thermo Scientific, Waltham, Massachusetts). The composition of the solution was the following: 7.5 mM zinc nitrate hexahydrate (Sigma-Aldrich, purum p.a., crystallized, ≥99.0% (KT)), 3.75 mM hexamethylenetetramine (Sigma-Aldrich, HMTA, ACS reagent, ≥99.0%), 0.10 M ammonia (Alfa Aesar, 28% v/v in water), 2 mM polyethylenimine (Sigma-Aldrich, PEI, ethylenediamine branched, average Mw ≈ 800, average Mn ≈ 600), 0.5 mM potassium chloride (Fluka, KCl, purum p.a. >99.0%), and 7.5 mM monoethanolamine (Sigma-Aldrich, MEA, reagent Plus ≥99.0%). The substrates were immersed in 80 mL of nutrient solutions. The reaction occurred for 24 h at 85 °C in a Termarks oven. The speciation plots to evaluate zinc, tin, and indium chemical species present in the solution were obtained from HySS, 4.0.31, Hyperquad Simulation, and Speciation software.^33^ Experiments were performed on control samples (seedless ZnO) and on samples (seeded ZnO) pretreated with a 0.5 mM potassium permanganate solution (Sigma-Aldrich, KMnO_4_, low in mercury, ACS reagent, ≥99.0%) at 90 °C for 20 min. Then, the samples were extensively rinsed in ultrapure water and sonicated (Elma SIOH Elmasonic bath system) for 10 min in ultrapure water.

### 2.2 Morphological and Spectroscopic Characterization of ZnO Nanostructures

*n*-ZnO deposited on ITO was characterized via a spectroscopic approach by means of diffuse reflectance and Fourier transform infrared (FT-IR) measurements. Diffuse reflectance spectra (DRS) of the *n*-ZnO samples were performed using a Jasco V-770 (Jasco, Tokyo, Japan) spectrophotometer equipped with an integrating sphere in the 300–800 nm wavelength range. The reflectance data were converted to absorbance based on Kubelka–Munk theory. Transmission mode UV–vis spectroscopic characterization of seeded and seedless ZnO was carried out by using a UV–Vis Specord S600 spectrophotometer (Analytik Jena, Jena, Germany) in the range of 300–650 nm. The analyses were conducted by using ITO/PET as blank to obtain the absorption spectra and its first derivative of *n*-ZnO. Cyclovoltammetric measurements to determine the electrochemical band gap were carried out in acetonitrile with 0.1 M tetrabutylammonium perchlorate (TBAClO_4_) as a supporting solution, using a Pt wire as the counter electrode and an Ag/AgCl (3 M KCl) electrode as the reference electrode. Attenuated total reflectance (ATR)-FT-IR spectra were recorded using an FT-IR Bruker 70 v Vertex Advanced Research Fourier Transform Infrared spectrometer (Bruker, Billerica, Massachusetts) equipped with an accessory ATR platinum and a diamond crystal, with 2 cm^–1^ steps and 120 scans in the acquisition range of 4000–70 cm^–1^. The spectrum of the empty crystal was used as the background and automatically subtracted from each sample’s spectrum. Static contact angle (CA) measurements were conducted using a DataPhysics Instruments SCA 20 optical contact angle measuring and contour analysis system (DataPhysics Instruments GmbH, Filderstadt, Germany), equipped with a CCD camera with high-resolution power, and applying the sessile drop method. For the CA measurements, 5.0 ± 0.5 μL droplets of ultrapure water were used, and the measurements were repeated three times for each sample. The topographical and morphological features of the samples were analyzed by collecting scanning electron microscopy (SEM) images of the samples with a TESCAN MIRA (TESCAN, Brno, Czech Republic) apparatus at 15 keV electron energy, 300 pA current, and a working distance of 9 mm. Before imaging, the samples were covered with a 20 nm thin gold film to improve the quality of the morphological analysis. The observed nanocubes (NCs) and *n*-ZnO densities were measured from SEM pictures using Fiji ImageJ software (version 1.53q). *n-ZnO* were counted, and their lateral sizes were measured from square areas selected from top-view SEM images; their densities were expressed as average counts per μm^2^.

The X-ray photoelectron spectroscopy (XPS) analysis was carried out using a ULVAC-PHI PHI 5000 VersaProbe II scanning microprobe (Chigasaki, Japan). Spectra were acquired using monochromatic Al Kα radiation (*hν* = 1486.6 eV) and a 100 μm diameter beam (50 W, 15 kV); electrons were collected at 45° with respect to the surface and analyzed with a hemispherical analyzer operating in the FAT mode. XPS depth profiles were realized by Ar^+^ sputtering (1 kV, rasterizing surface 2 mm × 2 mm), collecting a full spectrum every 12 s (0.2 min) of sputtering. All spectra were collected using a dual neutralization system (both e^–^ and Ar^+^ soft beams). X-ray diffraction (XRD) data were acquired by a Rigaku Smartlab SE XRD multipurpose diffractometer, using a Cu Kα radiation source (λ = 0.154 nm) run at 40 kV and 50 mA.

### 2.3 Electrical Characterization of ZnO Nanostructures

The electrical and impedance characterizations were carried out by employing a potentiostat/galvanostat instrument (Metrohm Autolab PGSTAT 128 N, Utrecht, Netherlands). The electrical response of the sensors was acquired as current–potential (*I–*V) analyses with a two-electrode connection (the two electrodes were the two ends of the ITO 50 mm × 5 mm active area connected by crocodile clips), short-circuiting the sensing electrode (S) with the reference electrode (RE), in order to avoid Ohmic losses, in the potential range of −2 to +2 V with a scanning speed equal to 0.1 V/s. The sensors were placed on an engineered folding device, designed through Fusion360 software and three-dimensional (3D) printed through stereolithography. The device is featured with a 10 mm sized curvature radius, which reports the angles used for bending. The angular measurements were verified through the use, in couple, of a digital protractor (Preciva) to validate the correspondence between the imposed angle and the angle actually measured; the angular measurement error is compatible with the accuracy of the digital goniometer (±0.3°). Bending measurements were conducted by fixing the sensors across the Kapton tape at both ends; in particular, one end was fixed with the Kapton tape, whereas the other one was varied so that the angular sliding corresponded to the translation of the sensor on the folding device. The samples were placed on the folding device, and their behavior was analyzed under static conditions at bending angles equal to 0, 30, 60, and 90°. At each bending step (i.e., from 0 to 30°), the folding device is elongated by approximately 5 mm. For each angle, 10 repeated measurements were performed. Chronoamperometric measurements were carried out to evaluate the time-dependent response of samples to bending by the same folding device. A +1 V bias was applied, and the current was measured by running a temporal scan continuously in an interval of 500 s, subjecting the samples to repeated bending cycles in the intervals 0°–90°–0°. Measurements of electrical resistance variations on wearable sensors were acquired by the PeakTech 2025 multimeter and extracted by the software DMM tool (version 2.0, PeakTech GmbH, Germany). Electrical impedance spectroscopy (EIS) measurements were carried out by employing a 3-electrode electrochemical cell. This setup includes the working electrode (active area 5 mm × 20 mm) consisting of ITO, seeded ITO, seeded ZnO and seedless ZnO, a reference Ag/AgCl (3 M KCl) electrode, and a platinum counter electrode, ensuring the current flow through the electrochemical cell. The three electrodes were immersed in an electrolytic solution comprising a phosphate-buffered saline (PBS) solution at pH 7.4 and investigated in a range of frequencies comprised between 100 000 and 0.1 Hz.

### 2.4 Photocatalytic Characterization of ZnO Nanostructures

The photocatalytic activity of the samples was evaluated in terms of the photodegradation of MB in an aqueous solution (3 mL, 25 μM). Irradiation was carried out using the Model 10500 ABET Low-Cost Solar Simulator (Abet Technologies, Old Gate Lane Milford, Connecticut) equipped with a 150 W Xenon arc lamp and an AM 1.5 G filter. The distance between the sample and the solar simulator was set using a calibration cell commercial KG5-filtered Si calibration cell (model 15151, Abet Technologies) to obtain an incident light power of 100 mW·cm^–2^ corresponding to 1 sun.

Before light irradiation, samples were soaked into the MB solution in the dark for 1 h to permit adsorption–desorption equilibrium. Irradiation effects were evaluated at 30 min intervals by analyzing changes in the absorption spectra of the MB solution. Spectra were acquired using a UV–vis spectrophotometer (Specord S600, Analitik Jena, Jena, Germany) in the range of 400–800 nm, with a wavelength accuracy of ±0.3 nm. Intensity measurements at 664 nm were allowed to quantify spectral changes. The pH of the MB solution was measured by using the Eutech pH 700 pH meter (Eutech Instruments Europe B.V., Landsmeer, Netherlands). The reusability of the seedless and seeded ZnO samples was tested by repeating the cycles of MB solution degradation on the same sample up to three times and by keeping the sample relaxed or under bending strain in a quartz cuvette. The sample was bent inside the cuvette, having an optical path of about 1 cm, maintaining an immersed area of about 30 mm × 5 mm. In the case of bending strain, the samples were folded inside the cuvette, keeping the two faces at a distance of about 2 mm. In between each photocatalytic test, the sample was washed with ultrapure water. The reproducibility was investigated by replicated photocatalysis experiments on three different seedless and seeded ZnO samples.

## 3 Results and Discussion

### 3.1 KMnO_4_ Seeding Effect on the ZnO Nanostructures Growth

**Scheme 1:**
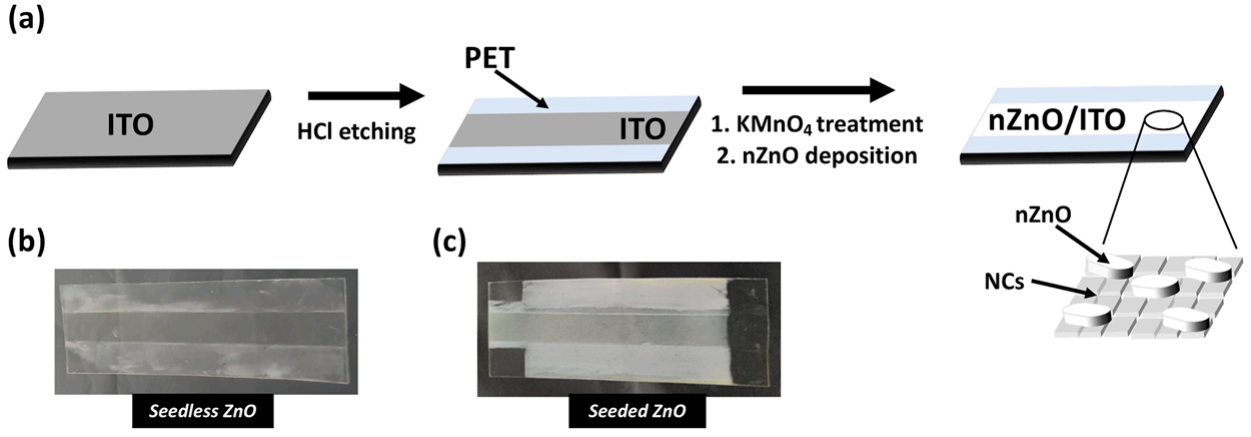
(a) Fabrication of the Photocatalytic Bending Sensor Onto ITO Electrodes, Following a Rational Approach Combining Nanocubes (NCs) Formed by Zinc, Indium, and Tin Oxides Onto Which *n-*ZnO Are Grown. Optical Photographs Showing the Two Devices: (b) Seedless ZnO and (c) Seeded ZnO *n*-ZnO grows on both the ITO exposed area and on the PET surface exposed after the HCl etching.

Scheme 1a reports the scheme used in this work for the realization of rationally designed *n-*ZnO onto ITO/PET. After the exposure of an active ITO area of 50 mm × 5 mm, the synthesis of *n-*ZnO on bare ITO or on seeded ITO was carried out following a previously optimized protocol for electronic interfaces at a mildly basic pH.^34^ The *n-*ZnO deposition conditions are expected to lead to a modification of the pH-sensitive ITO surface.^35^ Indeed, the basic pH of the zinc-rich nutrient growth solution induces the formation of the nanocubes (NCs) composed of indium, tin, and zinc mixed oxides as a result of the partial dissolution of ITO. Scheme 1b,c displays the optical photographic characterization of resulting samples realized in the absence (seedless ZnO) or in the presence (seeded ZnO) of the KMnO_4_ seed layer, respectively. As evident, the seed layer leads to a significantly higher amount of ZnO deposited on the ITO surface with respect to the seedless sample.

**Figure 1:**
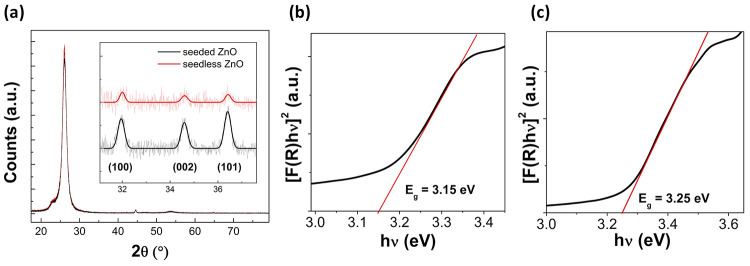
Characterization of seedless ZnO and seeded ZnO by (a) XRD, which shows the expected diffraction peaks of ZnO. DRS measurements highlighting the different energy bandgaps of (b) seedless ZnO and (c) seeded ZnO.

The study proceeded with an in-depth structural and morphological characterization of the different *n*-ZnO films formed with or without seeding. In particular, the crystallinity of the samples was evaluated by XRD measurements (see Figure 1a). As can be seen from the figure, the typical diffraction peaks of ZnO are found behind the peaks assigned to hexagonal wurtzite ZnO (JCPDS card No. 36–1451) without other impurity crystalline phases.^36^ Notably, the ratio of intensities from (100), (002), and (101) crystallographic planes highlights the different anisotropic growth of seeded and seedless *n-*ZnO. Indeed, for seeded ZnO, the (101) peak is the most intense, followed by the (100) and the (002) peaks, respectively. In the case of the seedless sample, the (100) peak is more intense than the (101) and the (002) peaks, respectively. The peak patterns of the seeded sample are in accord with previous reports dealing with 2D NSs,^37^ as well as ZnO NCs,^38^ showing a preferential (101) plane orientation and lacking a preferential orientation along the c-axis, which is typical of ZnO NWs. The higher (100) plane orientation of the seedless sample can be ascribed to the exposure of the lateral prismatic planes of the ZnO NFs.^39^ Interestingly, the crystallite domain sizes by the Scherrer formula^40^ for the seedless and seeded samples were 32.8, 26.3, and 29.9 nm for (100), (002), and (101) for seedless, respectively, whereas lower values were found for the seeded sample, being 25.6, 25.7, and 25.8 nm for (100), (002), and (101), respectively. The texture coefficients of the (002) and (101) crystallographic planes were calculated too.^40^ The resulting values for the (002) and (101) crystallographic planes were found to be 0.66 and 0.33 for seeded ZnO, and 0.61 and 0.89 for seedless ZnO, respectively. These data provide apparent evidence of the preferential growth of seedless ZnO and seeded ZnO around the (101) and the (002) crystallographic planes, respectively.

The presence of *n-*ZnO was further characterized by FT-IR spectroscopic analysis. In particular, FT-IR measurements showed the presence of the diagnostic Zn–O band at 540–570 cm^–1^ for seeded ZnO on the PET surface (see Supporting Information Figure S1), whereas no signal was obtained for seedless ZnO in the same experimental conditions. Also, it was not possible to observe the diagnostic ZnO signal on seeded or seedless ZnO grown on ITO/PET due to the absorption peaks of ITO. Interestingly, the presence of Mn in seeded ITO could be demonstrated thanks to the O–H bending vibrations combined with Mn atoms. Nevertheless, the exact attribution of these peaks is not straightforward, as they overlap with the same vibrational peaks found in the ITO sample (see Supporting Information Figure S2). To determine the optical band gap energy (*E*_g_) of the two different structures, UV–vis diffuse reflectance spectroscopy (DRS) characterization was carried out in the 300–800 nm wavelength range (see Figure 1b,c).^41^ The DRS data were transformed into the absorption coefficient by applying the Kubelka–Munk function *F*(*R*), and the values of *E*_g_ were estimated from the plot of [*F*(*R*)*hν*]^1/*n*^ versus the incident photon energy *hν* as the intercept of the extrapolation of the linear portion of curves to zero absorption on the *x*-axis.^42^ The acquired spectra for all of the investigated samples are reported in Figure S3. As ZnO is a direct band gap material,^43^ similar to previous reports on ZnO NSs and NFs,^28,44^ a value of *n* = 1/2, which accounts for the allowed direct transitions, was used. The obtained values were 3.25 eV for seeded ZnO and 3.15 eV for seedless ZnO. These *E*_g_ are lower than 3.37 eV, which is the typical value reported for bulk ZnO.^45^ This difference can be due to the size and morphology-dependent optical properties of the ZnO nanostructure,^46^ whereas manganese doping could determine a decrease of *E*_g_.^47^ Indeed, the *E*_g_ typically increases with a decrease in crystallite size of the ZnO nanostructure as a result of the optical confinement effect.^48^ Such confinement effects determine modifications such as the discretization of the energy levels and different density of states compared to the bulk, leading to shifts in the band gap.^49^ This is in accord with the herein reported experimental results, in which seeded ZnO corresponding to NSs show a higher *E*_g_ with respect to larger NFs. Such a decrease of *E*_g_ in comparison to bulk ZnO by morphological modulation can be highly beneficial for photocatalysis since it allows the use of less energetic and hazardous sources. Further characterization of seedless and seeded ZnO was conducted (Figures S4 and S5). UV–vis measurements conducted in the transmission mode allowed the evaluation of a band gap value of 3.5 eV, whereas it was not possible to determine a clear spectrum for seedless ZnO (Figure S4). Cyclovoltammetric measurements confirmed the difference between the valence/conduction energy levels between seeded and seedless ZnO by the determination of the reduction and oxidation onset potentials (see Figure S5). The resulting potential differences are equal to 2.74 and 2.67 V vs. Ag/AgCl (3 M KCl) for seeded and seedless ZnO, respectively. These values are equal to the electrochemical band gap.^50^ Importantly, the lower values of the electrochemical band gap with respect to the optical band gap can be ascribed to the presence of defects and trap states in *n*-ZnO.^51^ Nevertheless, these data are in accordance with the DRS data since they also show that seedless ZnO has a lower energy band gap than seeded ZnO.

Finally, water contact angle (CA) measurements (see Table S1 and Figure S6) were carried out to characterize the surface wettability of deposited *n-*ZnO. The measurements show that, as expected, the ITO surface is more hydrophobic after ZnO deposition, showing an increase from 65 ± 2° (washed ITO) to 75 ± 2° (ZnO onto ITO—i.e., seedless ZnO), respectively. A smaller increase is observed for seeded ZnO, i.e., from 82 ± 1° (seeded ITO) to 89 ± 2° (seeded ZnO). The obtained values for ITO and seedless ZnO are in good accordance with the values reported in the literature, being about 75 and 82°, respectively.^52,53^ The higher hydrophobicity of seeded ZnO and seedless ZnO vs. ITO can be likely justified by considering the increased surface roughness after NCs and *n-*ZnO deposition.^54^ Interestingly, the KMnO_4_ seed induces an increase in surface hydrophobicity compared to ITO. This is not expected since manganese oxide films should rather lead to an increase in hydrophilicity.^52^ Such a hydrophobicity increase can be again due to a modification in the surface morphology and subsequent roughness, in turn leading to an increase in the contact angle, and could also justify a minimum presence of manganese at the surface of ITO.

**Figure 2:**
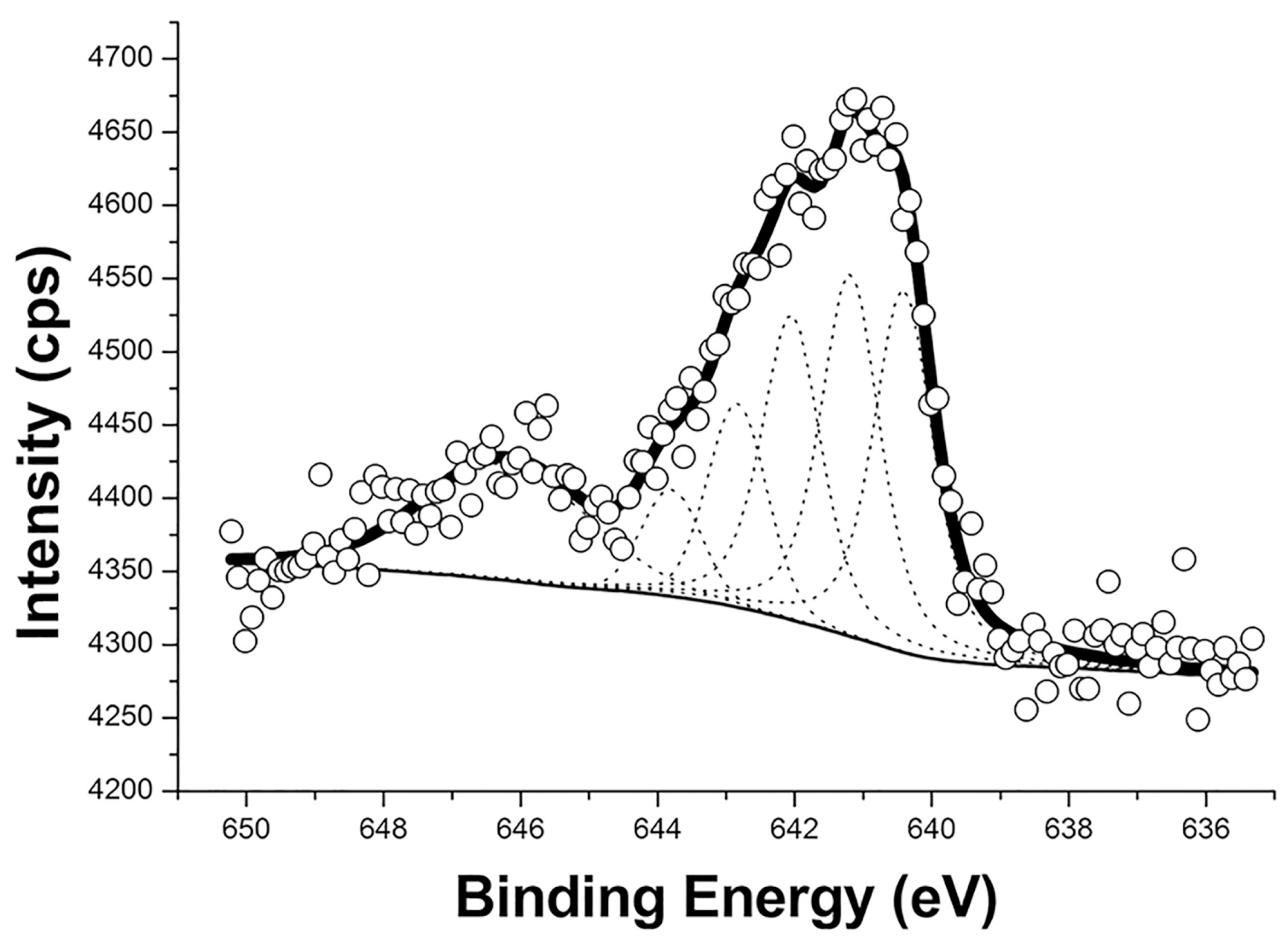
Mn 2p_3/2_ spectrum of the seeded ITO sample. The circles represent the collected XPS data, the thick solid curve is fit to the data, and the dashed curves show the diagnostic Mn(II) multiplet splitting and shake-up.

In order to address the superficial effect of seeding, the surfaces were analyzed by XPS. The overall observed effect of seeding may be seen as an increase of relative abundance of zinc on the surface (seedless composition, atom %: C 54.82, O 37.77, Zn 4.61, and In 2.80 and seeded composition, atom %: C 41.42, O 44.37, Mn 0.34, Zn 11.10, and In 2.77, considering that in both cases, the relative abundance of Sn is negligible). The relative abundance of the adventitious carbon may be misleading; a clearer picture may arise referring to the abundance of Zn with respect to a “constant” component, e.g., the ITO indium species. In fact, the Zn/In ratio goes from roughly 1.6 to 4.0 upon seeding; but the fact that Mn is still visible on the surface, though, is an indication of a nonperfect coverage of the ITO layer by the ZnO layer. By analyzing the surface before the ZnO crystal growth, it has been possible to observe some other effects. One of the direct effects of the MnO_4_^–^ treatment is the partial removal of adventitious carbon and other carbonaceous impurities from the surface. The comparison of the relative abundance of C in pristine ITO and treated (seeded) ITO shows a decrease in carbon content from 57 to 46 atom %. The removal of carbon by oxidation is further confirmed by the presence, on the treated surface, of residual reduced Mn(II). Angular profiles of the Mn 2p_3/2_ region showed linear dependence between the relative abundance and the sine of the take-off angle, suggesting the superficial confinement of such a residue. Moreover, it has been possible to analyze such a region in high resolution (see Figure 2), determining the presence of a diagnostic quintuplet and a prominent shake-up peak (646.20 eV; BE values are referred to adventitious carbon C–C/C–H peak at 284.80 eV, used as an internal reference) confirming that MnO_4_^–^ is reduced to Mn(II) upon seeding.^55^ Another remarkable effect of the seeding on the ZnO layer was revealed, though, by a depth profile analysis. It appears that, while in the seedless sample, it is possible to observe a distinct layer separation, ZnO relative abundance seems to be almost constant across the whole ITO layer, suggesting a crystalline growth, both forward and inward, in the ITO creeks.

**Figure 3:**
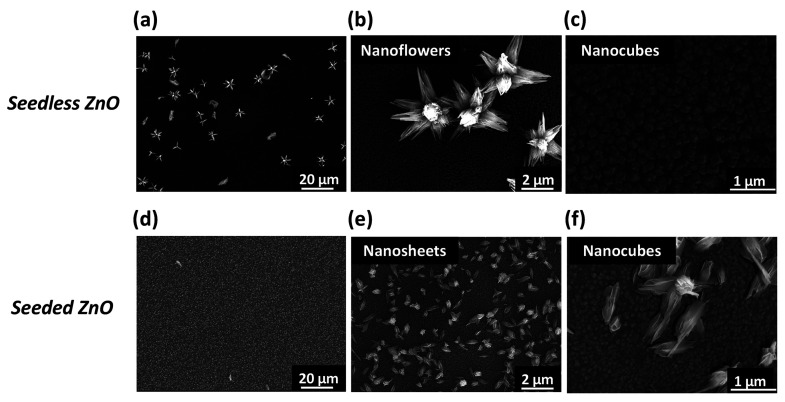
(a) Top-view SEM images of seedless ZnO showing sparsely distributed large-sized nanoflowers (NFs) and nanosheets (NSs). (b) Single NFs imaged on the ITO surface. (c) NCs observed in the areas not covered by NFs or NSs. (d) Top-view SEM images of seeded ZnO showing high-density smaller-sized NSs. (e) NSs observed on the ITO surface. (f) Single NCs observed in the areas not covered by NSs.

The structural characterization by SEM is reported in Figure 3. The surface morphology beneath *n-*ZnO is characterized by an almost regular pattern of nanocubes (NCs), having areas of side faces equal to 0.010 ± 0.001 μm^2^ and 0.005 ± 0.001 μm^2^ (average of 40 NCs) for seedless and seeded ZnO samples, respectively. Above the observed NCs, the *n-*ZnO morphology is finely controlled by the seeding induced by KMnO_4_. Seedless ZnO shows mainly NFs versus NSs, with a ratio of about 2.5 NFs per single NS. The surface coverage is about 10%, and *n-*ZnO appears disconnected (Figure 3a–c). The NFs are typically formed by an ensemble of nanoscale thin needle-like structures with lengths of about 3.6 ± 0.4 μm and widths equal to 0.6 ± 0.1 μm, respectively (average of 40 needles). The NS length is about 2.0 ± 0.6 μm (average of 14 needles). A completely different morphology is observed for seeded ZnO (Figure 3d–f). In this case, the surface appears more homogeneously covered (about 25%) and contains smaller nanoscale thin NSs with an average length equal to 0.6 ± 0.2 μm and width of 0.3 ± 0.1 μm (average of 40 NSs). Finally, the seeding induces an increase of about 20× times the number of *n-*ZnO on ITO.

The results of energy dispersive X-ray (EDX) elemental analysis performed with an accelerating voltage of 30 keV are reported in Table 1, from which it appears that the zinc amount in the seeded sample is about 5 times higher than the seedless. Although a marked difference in the Mn amount between the seeded and seedless samples would be expected, Mn was present at trace levels, near the minimum detectable limit. However, the Mn peak can be clearly observed in the EDX spectrum of the seeded ZnO sample (see Figure S7). EDX data from In, Sn, and Zn mixed oxide NCs in seeded and seedless ZnO samples are reported in Table S2.

**Table 1:** EDX Elemental Analysis of ZnO Seedless and ZnO Seeded Samples

atomic percentage values (atom %)	seedless ZnO	seeded ZnO
C	70.4	66.32
O	28.91	31.56
Mn	0.00	0.01
Zn	0.27	1.31
In	0.40	0.76
Sn	0.02	0.05

The NC formation in both seedless and seeded samples can be rationalized by considering the pH-sensitivity of the ITO surface,^35^ which, in turn, controls the morphology of the nanostructures grown on ITO. Indeed, ITO is composed of a mixture of two different oxides, In_2_O_3_/SnO_2_, both of them being amphoteric. In fact, they can be etched at both acidic^56^ and alkaline pH.^57^ Indeed, SnO_2_ is known to be stable up to pH 11,^58^ with Sn(OH)_5_^–^ and Sn(OH)_6_^2–^ being species released from SnO_2_ at pH slightly lower than this value. Similarly, In_2_O_3_ is slightly soluble at basic pH, with its solubility being on the order of 10^–5^ at a pH of 12.^57^ Accordingly, the basic pH used for the synthesis might lead to the release of tin and indium hydroxides, which can interact with zinc hydroxide species, leading to the formation of forming metal oxide nanocomposites. From previous studies, one can expect the formation of the metal oxide NCs. For instance, In(OH)_3_ can be a precursor of In_2_O_3_ NCs,^59^ whereas Zn(OH)_2_ and Sn(OH)_2_^2+^ can lead to the formation of the ZnO-SnO_2_ NCs.^60^

**Figure 4:**
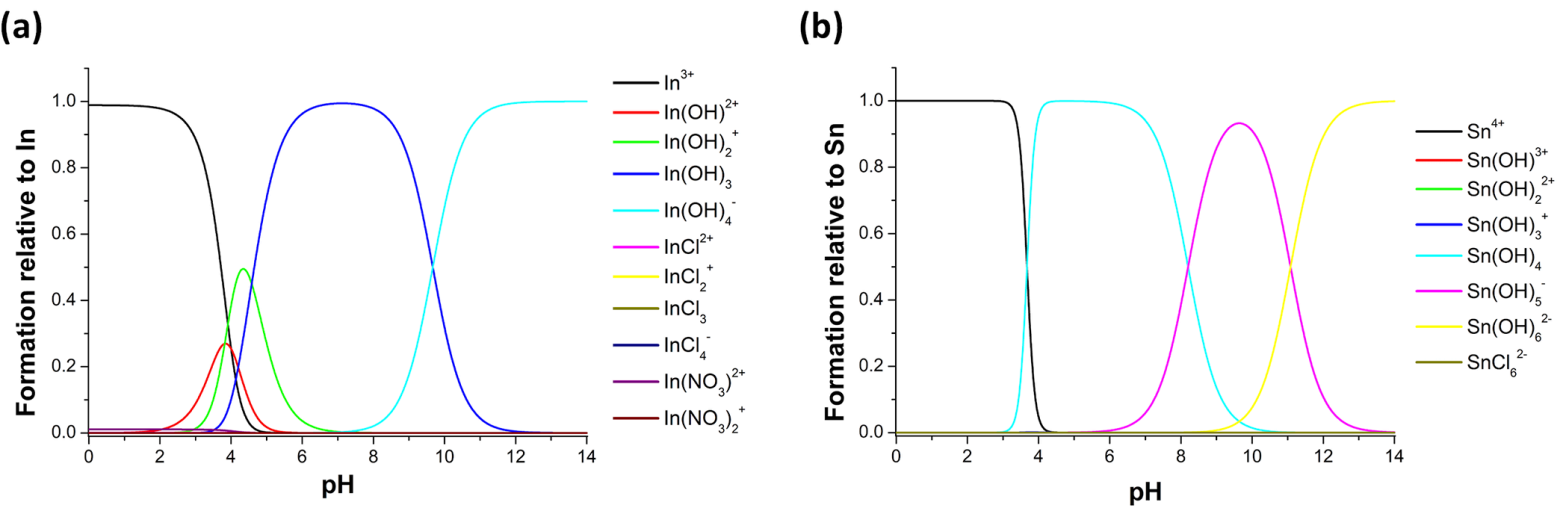
Speciation diagrams for (a) indium and (b) tin ions as a function of pH showing the formation of analytically relevant species.

With the aim to better understand the chemical species formed in the experiments, the analytical speciation of indium and tin ions as a function of pH is reported in Figure 4. From the speciation diagrams, at the basic pH of the nutrient growth solution (about 10), In(OH)_3_ and In(OH)_4_^−^ (see Figure 4a) along with Sn(OH)_5_^–^ and Sn(OH)_6_^2–^ species (see Figure 4b) are the most prevalent ones and could be released from the ITO surface, forming nanocomposites with zinc hydroxide species, such as Zn(OH)_2_. It can be expected that subsequent to the NC formation on the ITO surface, *n-*ZnO is deposited, given that the indium and tin hydroxides have lower concentrations with respect to the zinc species by moving away from the ITO surface.

### 3.2 Electrical Characterization

**Figure 5:**
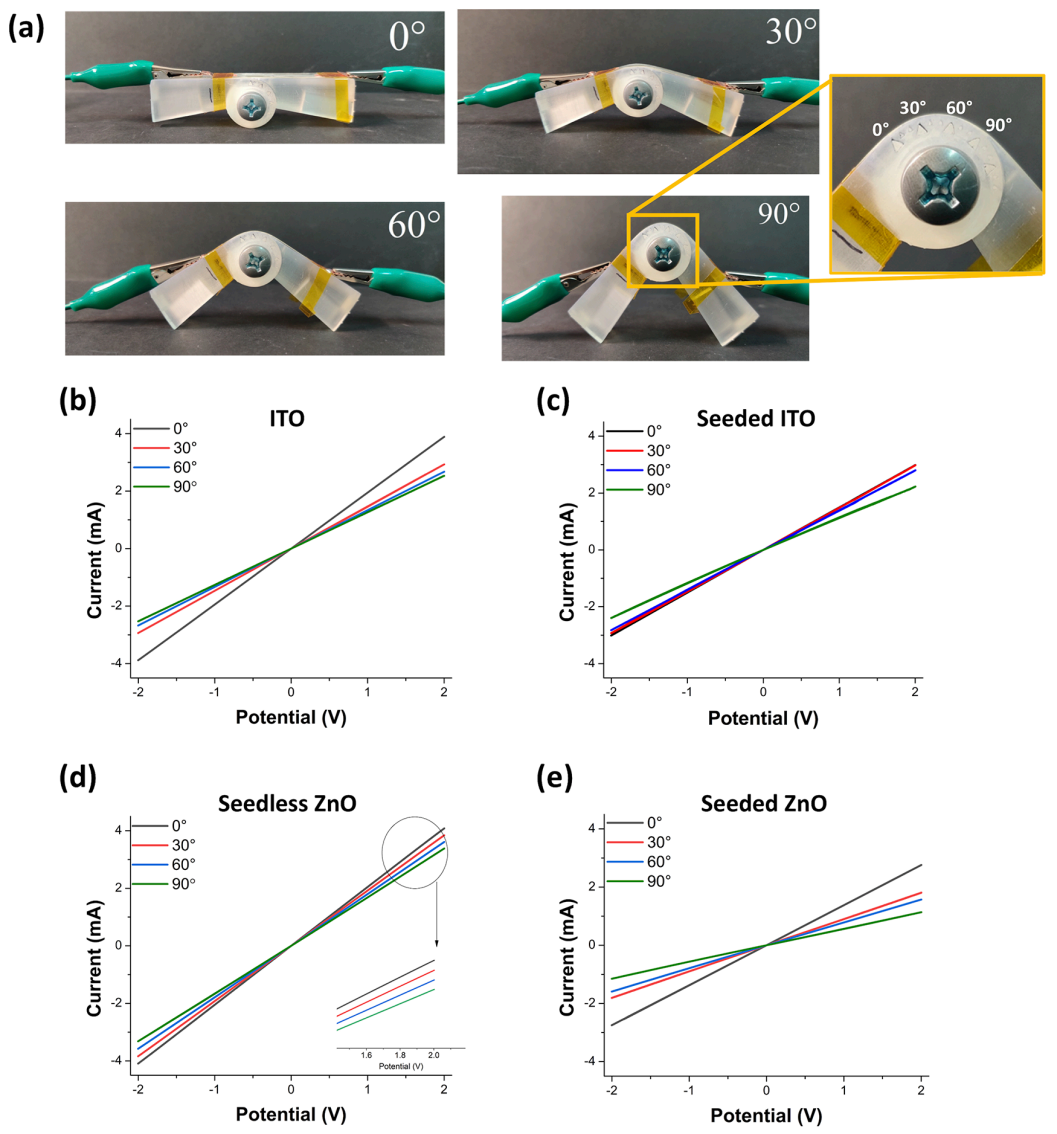
Electrical characterization of the devices. (a) Samples mounted on the 3D-printed engineered bending device at different bending angles (0, 30, 60, 90°). The inset shows the goniometer engineered on the bending device. *I*–*V* characteristic curves taken on (b) ITO, (c) seeded ITO, (d) seedless ZnO, and (e) seeded ZnO devices at different bending angles using the 3D-printed apparatus.

Figure 5 shows the setup employed for evaluating the electrical responses of *n-*ZnO under bending. The devices were mounted on the 3D-printed apparatus (see Figures 5a and S8) with one end attached and the other one set free for bending at fixed angles (0, 30, 60, and 90°) at a bending radius of 10 mm, allowing the extraction of the *I*–*V* curves. Bending clearly induced a decrease of the current slope for the investigated samples. The ITO control samples (see Figure 5a,b) showed a current variation (about 0.2 mA) in the 30–90° bending angle range. A similar trend was found for the seeded ITO sample. Interestingly, seedless ZnO (Figure 5b) showed current values similar to those of ITO and higher than seeded ZnO (Figure 5c), which can be ascribed to the higher amount of *n*-ZnO in the latter system. In particular, seedless ZnO showed a regular decrease of about 0.3 mA per each angle, while the decrease for seeded ZnO from 0 to 30° was 0.9 mA, and about 0.4 mA for the variations from 30 to 60° and finally 90°. A statistical analysis (average of 10 different measurements) of the decrease of current values measured at +2 V was also performed (Figure S9).

Subsequent to the initial characterization by *I*–*V* curves, chronoamperometric measurements for bending in the interval 0 to 90° under +1 V bias were carried out to investigate the dynamic responses of the sensors and to evaluate their repeatability, i.e., whether current variations were kept constant over time. Accordingly, the observed decrease in the Δ*I* amplitudes was in the range of about 20–30 μA for seedless samples, showing an initial peak at about 60 μA, whereas the respective increase for seeded ZnO was as high as 200 μA (Figure S10). It is noteworthy that the noise in the current signal observed in seeded ZnO could be ascribed to the disturbances induced in the electric contacts by the bending setup when the strain was applied. Control experiments on ITO devices showed a much lower response to bending under the same experimental conditions, with the Δ*I* amplitudes being in the range of a few μA (see Figure S10). Indeed, the significantly higher decrease in Δ*I* observed for seeded and seedless ZnO in comparison to ITO might be ascribed to the piezopotential generated due to the deformation of *n-*ZnO by the bending strain, whereas a purely piezoresistive mechanism takes place for ITO. Nevertheless, the different morphology of NCs in seeded ZnO and seedless ZnO might well induce piezoresistive effects that can significantly contribute to the different electrical responses, in accordance with previous reports.^61^ For the *n-*ZnO-based devices, bending induces a clear decrease in current, while no further decrease is observed during the time interval when the applied bending is constant. The current returns to the initial value once the bending stress is removed. The results of these electrical characterizations (i.e., a decrease of Δ*I* amplitudes upon bending) are in agreement with previous reports illustrating that the operative mechanisms of high-density ZnO NW bending sensors were realized onto graphene electrodes.^62^ These reports show that a downward bending produces a compressive strain applied to the ZnO NWs, causing the formation of holes at the ZnO NWs/graphene interface, which ultimately leads to the observed current decrease. The piezoelectric response originated from the well-known piezoelectric properties of 2D *n-*ZnO, which are also reported to outperform ZnO NWs.^37^ However, one has to consider that piezoresistive effects resulting from the NC morphology can induce electrical discontinuities and voids in the film, thereby producing a significant electrical response under bending, in accordance with models dealing with piezoresistive materials.^63^ The strain induced by bending on the devices was calculated using the formula ε = *h*/2*R*, where *h* is the thickness of the substrate (about 150 μm for ITO/PET) and *R* is the bending radius. The curvatures for bending angles of 30, 60, and 90° are equal to 16, 14, and 12 mm, respectively. It is possible to estimate an average gauge factor  of the sensors, where ε, Δ*R*, and *R* are the strain applied to the sensor, resistance variation at ε strain, and initial resistance at zero strain, respectively. The average gauge factor was ∼160 for seeded ZnO compared with ∼25 for seedless ZnO, along with the control values of 75 and 30 for ITO and seeded ITO, respectively. The seeded ZnO sensor outperforms the other ones tested in this work and is comparable to the best ones reported in the same average experimental conditions for similar devices based on *n*-ZnO grown on PET.^62^

**Figure 6:**
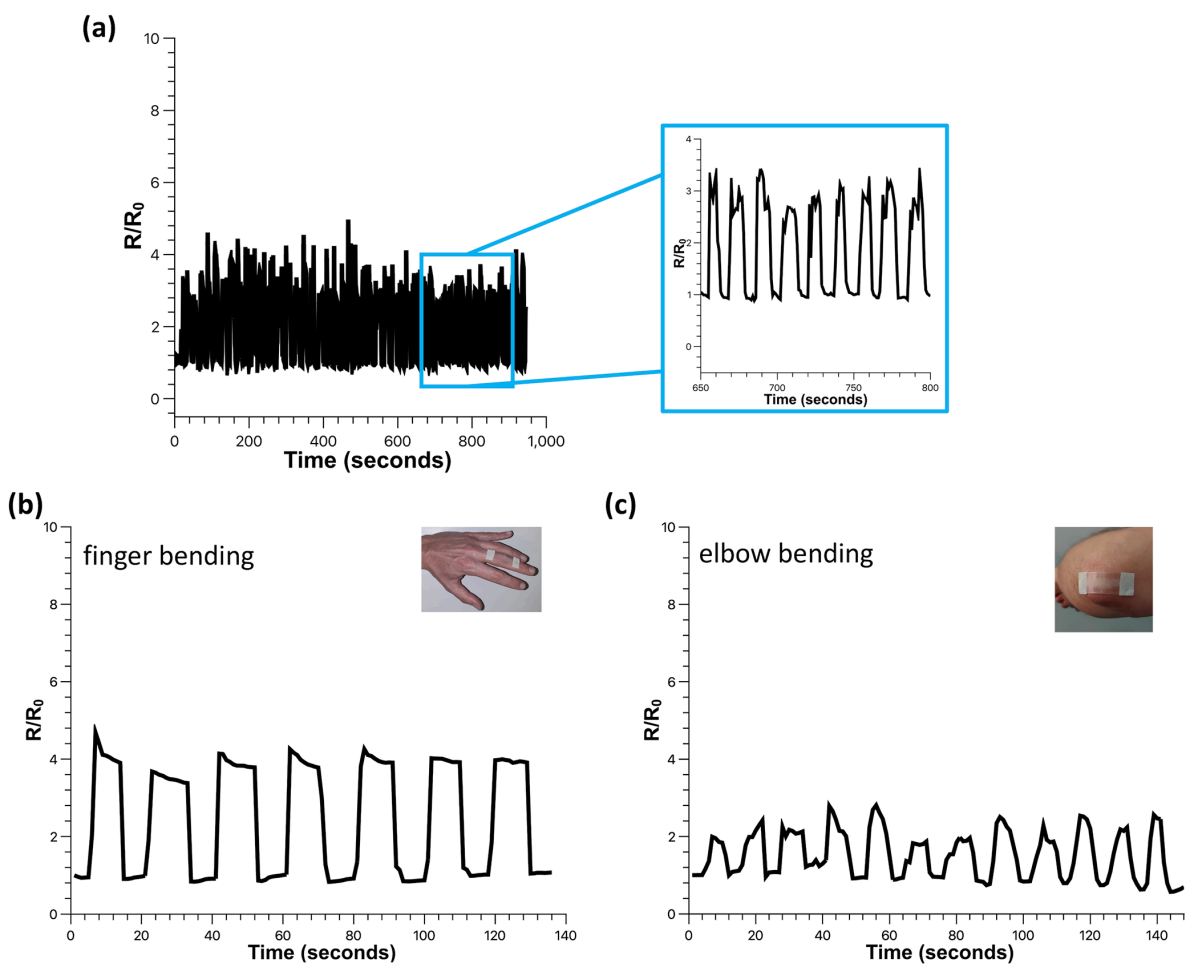
(a) Electrical resistance response of multicycle 0–90° bending of the ZnO-seeded sensor. Real-time monitoring of the (b) finger and (c) elbow bending.

To show the promising potentialities in functional devices, as shown in Figure 6a, the detection of resistance variation of the seeded ZnO sensor undergoing a series of multiple 0–90° bending tests demonstrates the repeatability and durability of the sensor under repeated stress conditions. The sensors can be easily worn, permitting continuous monitoring of the bending–release motion of the finger (Figure 6b) and the elbow (Figure 6c), enabling applications in wearable devices.

**Figure 7:**
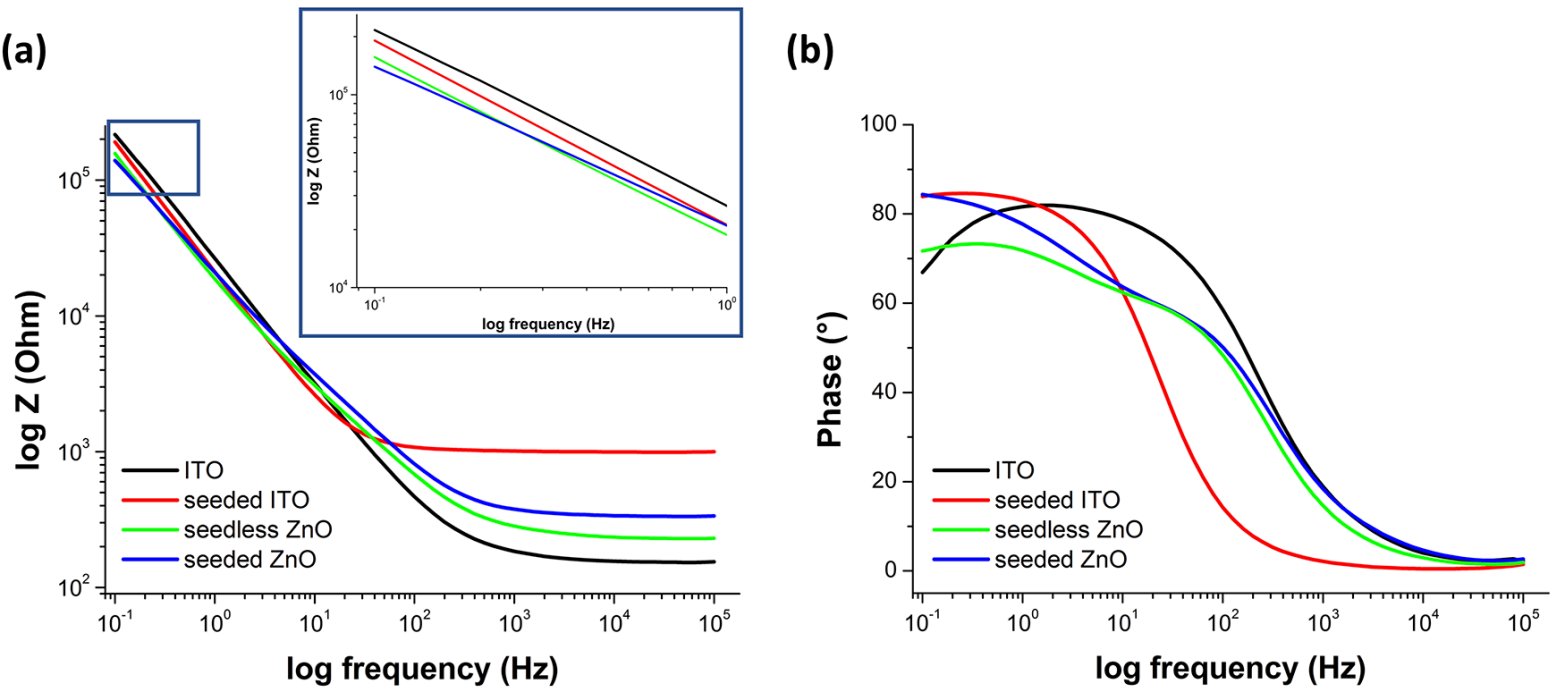
Electrochemical impedance spectroscopy characterization. A comparison of seeded ZnO and seedless ZnO with respective control samples by means of (a) the measured impedance in PBS (pH 7.4) as a function of frequency and (b) the oscillation phase as a function of frequency. Inset shows the impedance values at low frequencies.

Electrochemical impedance spectroscopy (EIS) measurements in PBS buffer (pH = 7.4) (see Figure 7) allowed us to estimate the magnitude of the interfacial layer resistance produced by the electrode surfaces. The electrode/PBS electrolyte interfaces are described as in parallel resistors/capacitors, which are in series with the PBS solution resistance. The values of impedance (Figure 7a) and oscillation phase (Figure 7b) as a function of frequency highlight a different behavior for seedless ZnO and seeded ZnO vs. the respective control samples. At high frequencies (10^3^–10^5^ Hz), the phase angle measured is almost 0°, indicating a purely resistive diffusion-limited electrolyte motion to the electrode. In contrast, at frequencies lower than 10^3^ Hz, an increase of the capacitive component of the electrode/electrolyte interface is observed due to the fact that the resistance component of the electrode/electrolyte interface is dominant with respect to both the solution resistance and the impedance of the parallel capacitive component. Different from the ITO surface, for which a decrease in phase is observed at frequencies lower than 1–0.1 Hz, the ZnO samples maintain a slightly higher capacitive behavior. In addition, the impedance values of the seeded ZnO samples are lower than seedless ZnO and significantly lower than ITO, which can be ascribed to the higher surface exposure of *n-*ZnO with respect to the ITO electrodes. Nevertheless, the value of the ZnO impedance is correlated to the morphology of *n-*ZnO, with NSs being, in particular, able to reduce the impedance with respect to NWs.^64^ As a result, *n-*ZnO permits a significant reduction of the interfacial layer resistance, decreasing, in turn, the surface charge processes, which are essential in photocatalysis.

### 3.3 Photocatalytic Activity

**Figure 8:**
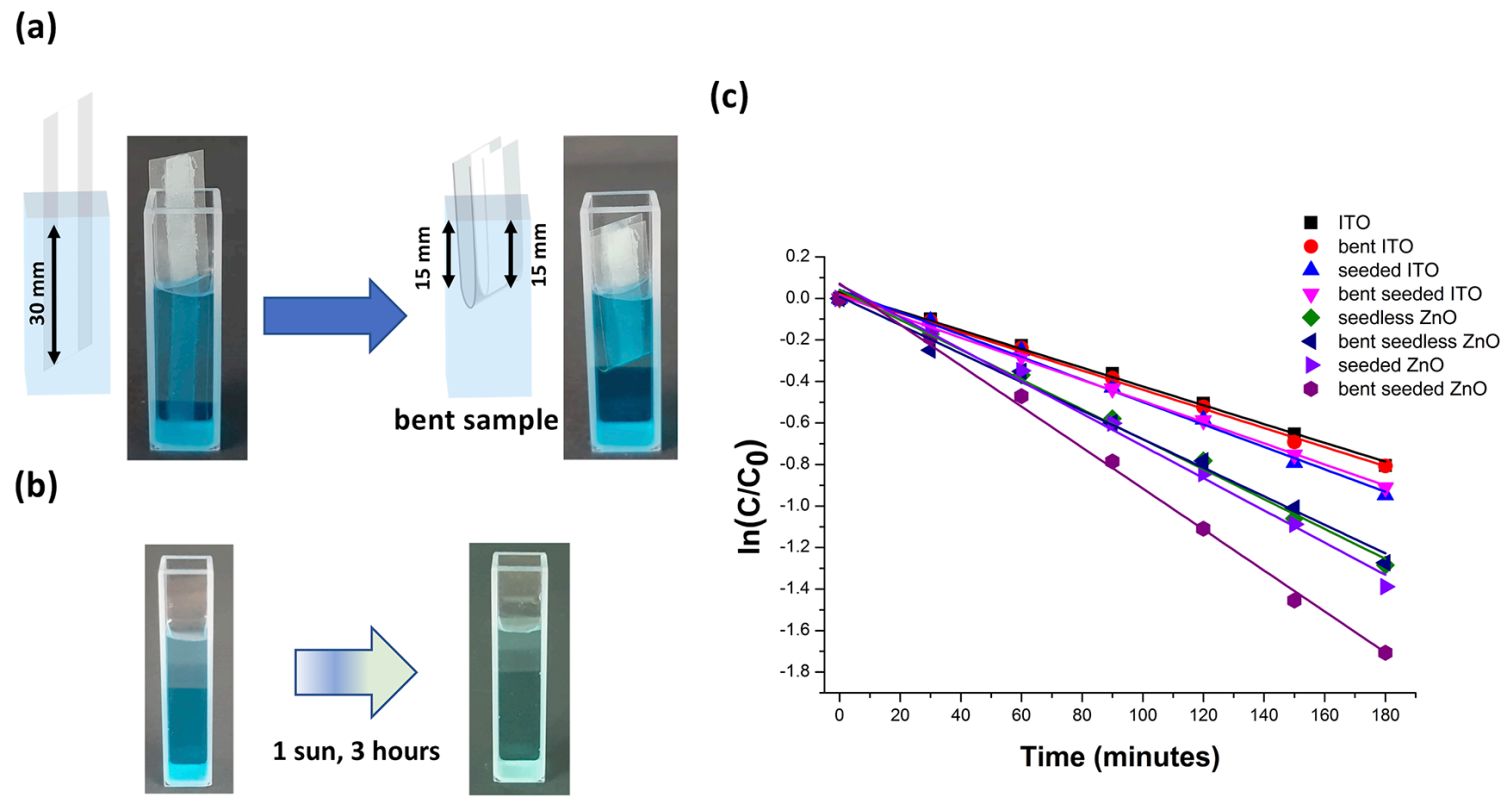
Photocatalytic degradation of the MB dye. The samples were immersed into a quartz cuvette containing the MB solution (25 μM) and kept in the dark for 1 h to allow for dye adsorption. (a) Photocatalytic activity determined by keeping the sample undeformed or folded inside the cuvette. (b) Optical investigation of the discoloration resulted from a photocatalytic cycle. (c) Kinetic curves of MB degradation under 1 sun obtained for the investigated samples. Only the seeded ZnO sample shows a significant increase of photocatalytic efficiency upon bending.

Experiments of photocatalytic degradation of MB were carried out on both seedless and seeded ZnO. In Figure 8, the measurement setup is shown. The samples were immersed in a quartz cuvette containing 3 mL of an MB solution at 25 M concentration (*C_i_*) and kept in the dark for 1 h to reach the adsorption–desorption equilibrium prior to the catalytic process. The samples were immersed as unmodified or bent inside the cuvette (see Figure 8a) to test the effect of strain on the photocatalytic performances. The immersed area was equal to about 30 mm × 5 mm in both cases. The MB adsorption efficiency was scarce after 1 h, except for the bent ZnO-seeded sample. The cuvette was irradiated using a solar simulator, resulting in the discoloration of the MB solution (see Figure 8b). The use of a light source offering similar intensity and spectral composition to natural sunlight is fundamental to testing the photocatalytic efficiency of the investigated systems under indoor conditions simulating real-life scenarios. The MB photodegradation kinetics was evaluated by monitoring the decay of absorbance intensity at λ = 664 nm, as already shown in our previous work.^65^ As reported in Figures S11 and S12, with the increase of irradiation time, the decrease of the absorption peak intensity was observed in the investigated samples, i.e., ITO, seeded ITO, seedless ZnO, and seeded ZnO, in the absence or in the presence of bending. It was found that the photocatalytic degradation rate can be modeled by a pseudo-first-order kinetic model in which the rate constant (*k*) is evaluated by the linear regression model expressed by ln(*C*/*C*_0_) = *kt*, where *C*_0_ is the concentration after adsorption/desorption equilibrium and *C* is the concentration after a given exposure at 1 sun irradiation time of the MB aqueous solution (Figure 8c). The MB photodegradation in 180 min for seeded ZnO was quite similar with respect to seedless ZnO, with the photodegradation being equal to 71 ± 3 and 70 ± 1% (average values of three different samples), respectively. Albeit the higher concentration of *n-*ZnO on the seeded sample compared to the seedless, the lower impedance, the slight predominance of (101) orientated crystallographic planes known to be photoactive,^39^ as well as the lower band gap value of *n-*ZnO of the seedless sample, can counterbalance these effects, ultimately leading to similar efficiencies. The apparent reaction constants *k* for seedless ZnO and seeded ZnO were 0.0072 ± 0.0002 and 0.0077 ± 0.0003 min^–1^, respectively. These have to be compared with the values from control experiments, i.e., MB solution, ITO, and seeded ITO, which reach 0.0049 ± 0.0002, 0.0045 ± 0.0002, and 0.0054 ± 0.0002 min^–1^, respectively (see Figure S13 in the Supporting Information). These data indicate that control ITO samples do not significantly increase the photocatalysis of MB in solution, whose photodegradation kinetics is well in accordance with previous reports.^14^ Bending did not produce a significant effect on the photodegradation in seedless ZnO (0.0067 ± 0.0003 min^–1^), ITO (0.0046 ± 0.001 min^–1^), and seeded ITO (0.0049 ± 0.0002 min^–1^). A mild improvement was observed only for seeded ZnO (0.0099 ± 0.0003 min^–1^), with its kinetic constant being about 25% higher than the rate constant in the absence of bending. The reusability of the samples was tested by repeating the cycles of MB solution degradation on the same sample up to three times, in the presence or in the absence of bending. In between each photocatalytic test, the sample was washed with ultrapure water. The seedless ZnO samples show excellent reusability under up to three cycles, both in the absence or in the presence of bending. In particular, the photodegradation efficiencies for the seedless ZnO sample were equal to 71% (first cycle), 74% (second cycle), and 67% (third cycle). In the case of bending, the photodegradation efficiencies were equal to 71% (first cycle), 72% (second cycle), and 70% (third cycle). It is clear that bending does not affect nor induce ameliorative effects in the photodegradation efficiencies. Similarly, the seeded ZnO samples maintained optimal reusability up to three cycles in the absence of bending, given that the photodegradation was equal to 72% (first cycle), 74% (second cycle), and 78% (third cycle). A different scenario was observed in the case of the seeded ZnO samples under bending. In fact, the bent seeded ZnO sample shows a clear increase of photodegradation in the first (81%) and second cycle (80%), whereas a decrease of photodegradation in the third cycle is observed (69%), reaching values similar to those in the absence of bending. This decrease of efficiency might be ascribed to the dissolution of ZnO into the solution phase induced by bending or to the reduction of the available interface due to MB adsorption on the ZnO surface. The pH value of the MB solution was measured in the range of 7.6 ± 0.2, which is only slightly lower than the one after photodegradation (7.3 ± 0.3). This pH value is not acidic enough to favor the complete dissolution of *n*-ZnO, even though partial degradation can still occur.^23^ Control experiments (see Figures S14 and S15) conducted in the absence of light stimulation demonstrate that seeded ZnO shows a minimal decrease (about 3%) in the MB concentration after 180 min, which is not very different from the 6–7% increase of MB photodegradation upon bending and solar light. The higher degradation value might be ascribed to the higher wettability of *n*-ZnO upon solar light exposure^66^ with respect to the dark environment. This can, in turn, favor MB adsorption on the active surface and hence enhance photocatalysis. Nevertheless, the dim increase of MB degradation under bending in the dark is in accordance with previous reports based on ZnO NWs,^67^ where the piezoelectric field generated by pure mechanical strain mainly plays only a supporting role in the piezophotocatalytic process. In addition, it should be considered that the eventual piezopotential created by *n*-ZnO in the aqueous environment attracts ions toward its surface, finally compensating the piezoelectric charge.^68^ This leads to a significant decrease of piezoinduced potential usable in the piezophotocatalytic process with respect to the one generated in the solid-state bending sensor. Indeed, this observation might explain the reason why, in the case of seedless ZnO, no piezoinduced enhancement in the photocatalytic effects is observed. The photocatalytic performances of the *n*-ZnO sensors are well in agreement with the results of previous reports, which are shown in Table S3.

## 4 Conclusions

Herein, a rational approach to control the *n*-ZnO synthesis onto flexible ITO/PET is shown, resulting in bending sensors with reconfigurable photocatalytic performances. The basic pH used for the ZnO synthesis enables the formation of NCs, resulting in composites formed by zinc, indium, and tin. Onto them, the *n*-ZnO growth is controlled by KMnO_4_ seeding that enhances the density of *n*-ZnO to >20-fold value. Although low-density NFs and NSs are grown on the untreated sample, the seeded sample favors the formation of smaller, high-density 2D NSs. The optical and electrical characteristics of the two systems allow for the realization of bending sensors that combine good sensitivity toward bending in combination with excellent photocatalytic properties, finding that the seeded sample bearing 2D NSs shows the best performances. Future work will deal with a systematic investigation of photocatalysis as a function of different angles/strains to elucidate the occurrence of a piezophotocatalytic process. Finally, this work provides an innovative insight into the role of controlling *n*-ZnO structural/functional properties in the combination of bending sensing and photocatalytic properties, paving the way toward eco-friendly devices by rational materials morphology engineering.

## Supporting Information Available

FT-IR, UV−vis, and EDX characterization of the materials, cyclic voltammetric analysis, surface energy estimation, and electrical and photocatalytic characterizations (PDF)
