# Supplementary material for: Self-Cleaning Bending Sensors Based on Semitransparent ZnO Nanostructured Films
Source: ACS Appl Eng Mater. 2023 May 3;1(5):1384–96. doi: 10.1021/acsaenm.3c00082 (PMC10226038; doi:10.1021/acsaenm.3c00082)
Supplement: Supplementary file 1 — em3c00082_si_001.pdf [file em3c00082_si_001.pdf]

Supporting Information

Self-cleaning Bending Sensors Based on Semitransparent ZnO Nanostructured Films

Giuseppe Arrabito,[a, ‡] Antonio Delisi,[a,‡] Giuliana Giuliano,[a] Giuseppe Prestopino,[b] Pier Gianni Medaglia,[b] Vittorio Ferrara,[a] Federica Arcidiacono,[c] Michelangelo Scopelliti,[a,d] Delia Francesca Chillura Martino,[c,d] Bruno Pignataro[a,d]**

[a] Department of Physics and Chemistry - Emilio Segrè, University of Palermo, Viale delle Scienze 17, 90128 Palermo, Italy
[b] Department of Industrial Engineering, University of Rome “Tor Vergata”, Via del Politecnico 1, 00133 Rome, Italy
[c] Department of Biological, Chemical and Pharmaceutical Sciences and Technologies (STeBiCeF), University of Palermo, Viale delle Scienze 16, 90128 Palermo, Italy
[d] National Interuniversity Consortium of Materials Science and Technology (INSTM), UdR of Palermo, 50121 Florence, Italy

‡ G.A. and A.D. contributed equally.

*To whom correspondence should be addressed. E-mail: giuseppedomenico.arrabito@unipa.it; bruno.pignataro@unipa.it

Table of Contents

1. **FT-IR analysis**
2. **UV-vis characterization**
3. **Cyclic voltammetric analysis**
4. **Surface energy estimation**
5. **EDX characterization**
6. **Electrical characterization**
7. **Photocatalytic characterization**

1. FT-IR analysis

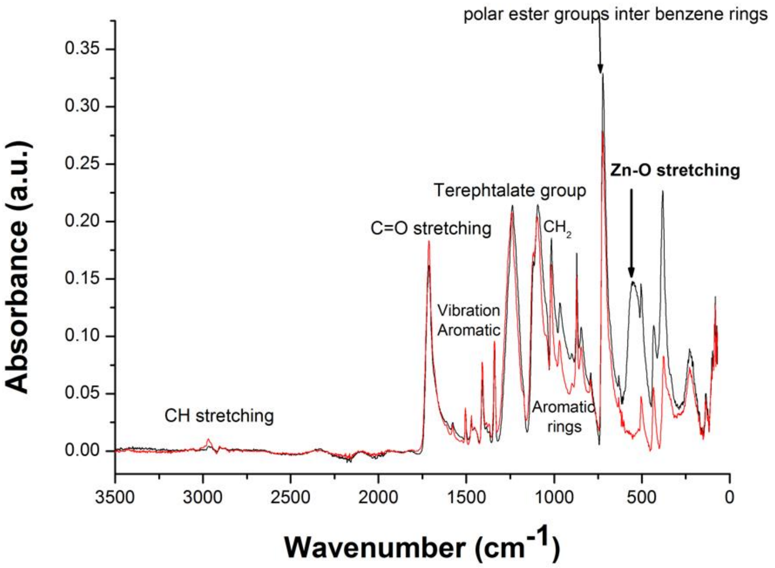

Figure S1. FT-IR characterization of the nZnO deposited on PET in the presence of $KMnO_4$ seeding (red line) in comparison to bare PET (black line).

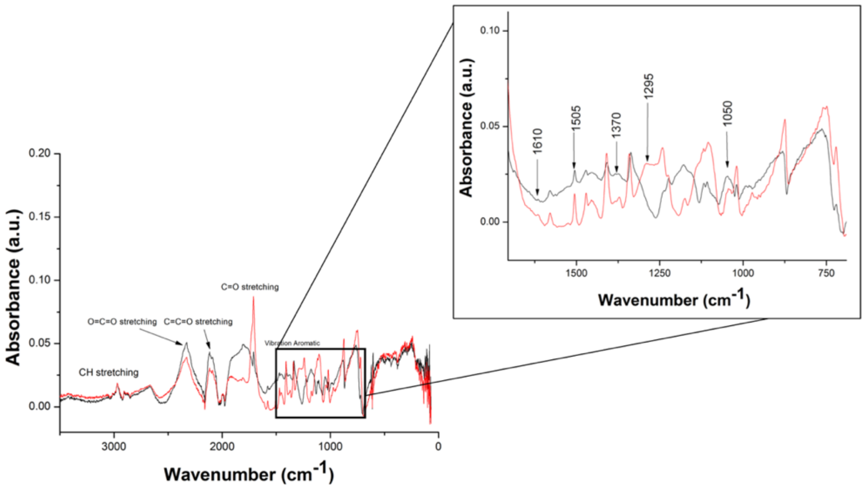

Figure S2. FT-IR characterization of ITO after $KMnO_4$ seeding (red line) in comparison to bare ITO (black line). The inset on the right shows the region comprised in the region between 700-1700 cm^{-1}.

It was not possible to detect the Zn-O diagnostic peaks (wavenumber range between about 540-570 cm^{-1}) on ITO both for seedless and seeded ZnO samples. This is due to the high background signal of the ITO and the presence of the In-O peak[1] at 430 cm^{-1}. Differently, the analysis on the PET

portion of the seeded ZnO sample (**Figure S1**) permitted to identify the characteristic absorption peak falling in the wavenumber range between about 540-570 cm^{-1} typical of Zn-O bond stretching.[2] Along with it, the typical absorption peaks of PET were identified in accordance with previous results.[3] Specifically, the peak at 712 cm^{-1} is ascribed to the interaction of polar ester groups and benzene rings. The peaks attributed to the aromatic rings are found in the region between 972, 872 and 848 cm^{-1}. The absorptions due to methylene groups and vibrations of the ester bond (C-O) are found within the absorption band between 1096 cm^{-1} and 1050 cm^{-1}. The absorption peaks around 1240 cm^{-1} and 1124 cm^{-1} can be attributed to the terephthalic group. It is possible to observe at a wave number of about 1730 cm^{-1} the presence of the stretching of the carboxylic acid group (CO). In the region between 2900 cm^{-1} and 3000 cm^{-1}, the absorption peak characteristic of CH stretching is visible. The presence of Manganese in the seeded ITO (see **Figure S2**), already proved by XPS analysis (see **Figure 2** in the main text) could be demonstrated thanks to diagnostic bands attributed to O–H bending vibrations combined with Mn atoms [4,5] in the range 1000-1600 cm^{-1}, namely at the peaks (1610 cm^{-1}, 1370 cm^{-1}, 1050 cm^{-1}). However, the exact attribution of these peaks is not straightforward being close to the peaks observed for the ITO sample. Intriguingly, the peaks at 1505 cm^{-1}, 1295 cm^{-1} and 1050 cm^{-1}could be ascribed to hydrous MnO_2 as reported in reference 5.

2. UV-vis characterization

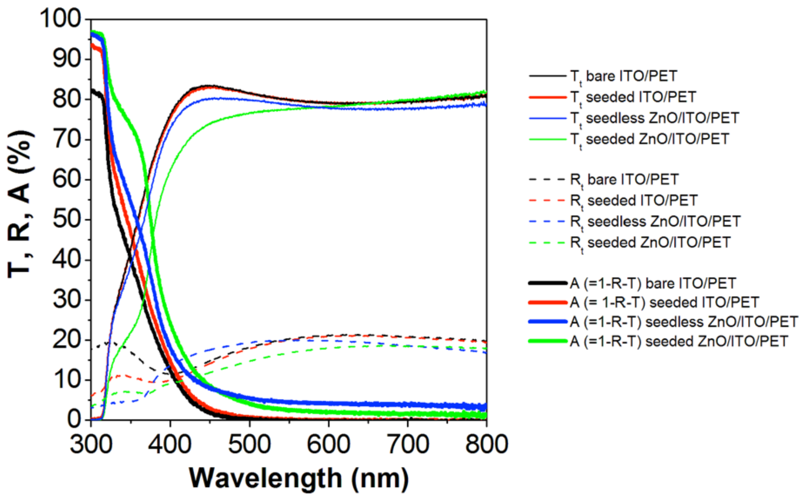

Figure S3. UV–Vis Diffuse Reflectance Spectroscopy (DRS) characterization on the samples.

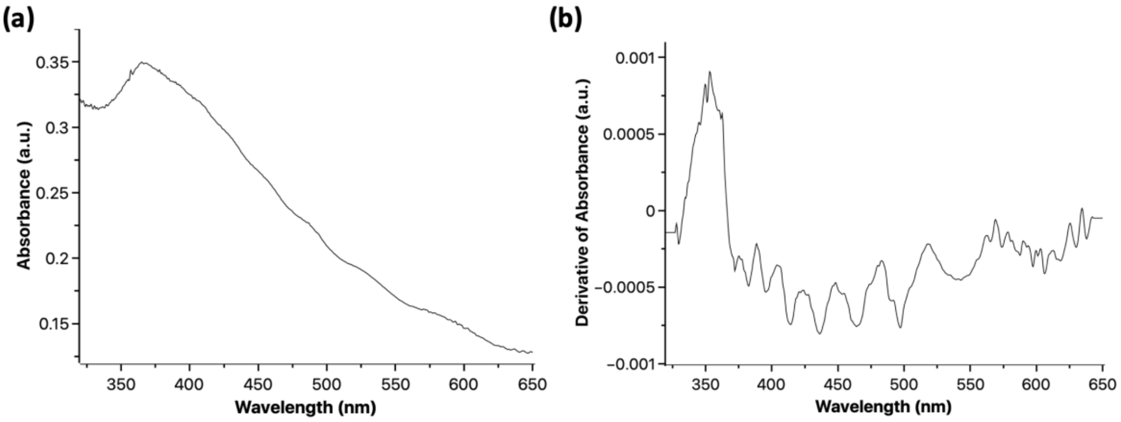

Figure S4. **(a)** Transmission UV-vis spectra of seeded ZnO and **(b)** first derivative of the absorption spectrum.

3. Cyclic voltammetric analysis

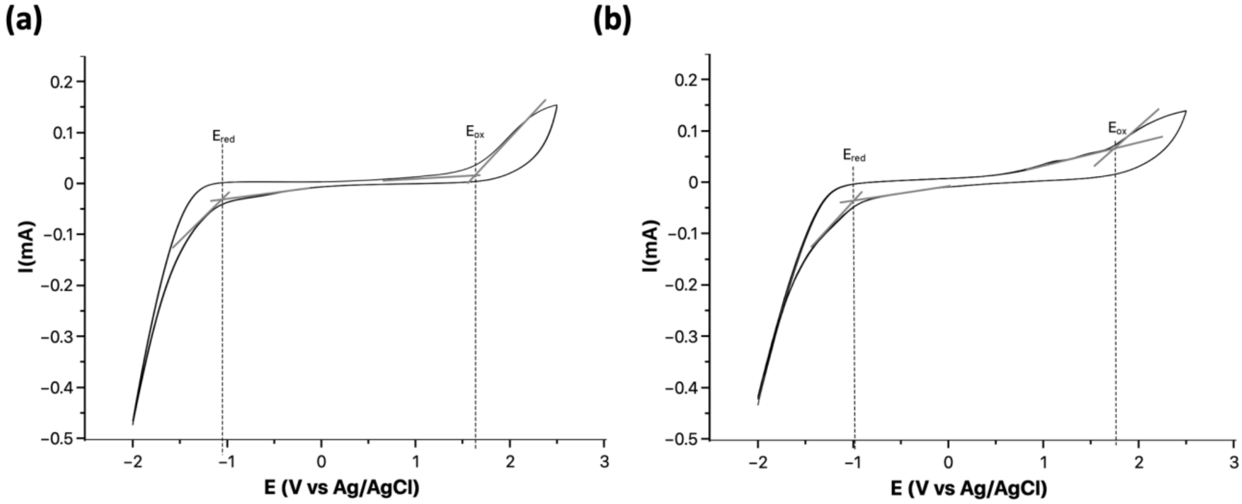

Figure S5. Cyclovoltammetry measurement of **(a)** seedless ZnO **(b)** seeded ZnO electrodes acquired on in acetonitrile containing 0.1 M $TBAClO_4$. Scan rate, 200 mV/s. The dashed lines represent the onset reduction (E_{red}) and oxidation (E_{ox}) potentials.

4. Surface energy estimation

A rough estimation of the surface free energy (γ_s) of the examined solid surfaces was obtained from the single contact angle measurements using the model proposed by Neumann et al. [6]:

$$cos(\theta) = -1 + 2\sqrt{\frac{\gamma_s}{\gamma_l}}e^{-\beta(\gamma_s-\gamma_l)^2}$$

where γ_s is the solid free surface energy, γ_l is the liquid free surface energy (for water γ_l = 72.7 mJ/m^2, [7]), θ is the contact angle, and β is an empirical constant with an average value of 0.0001247 (m^2/mJ)^2. The resulting values are listed in Table S1. Notably, the values obtained for PET and ITO are in the range of the values which can be found in the literature using other models as well.[8,9]

Table S1. Static contact angle values of water θ_w **(°)** and different values of surface energy γ_s (mJ/m^2) of the different investigated samples. The reported results were obtained as the mean of three CA measurement for each sample.

	PET	ITO/PET	seeded ITO	seedless ZnO	seeded ZnO
**θ_w (°)**	74 ± 1	65 ± 2	82 ± 1	75 ± 2	89 ± 2
γ_s (mJ/m^2)	39 ± 1	44 ± 1	34 ± 1	38 ± 1	30 ± 1

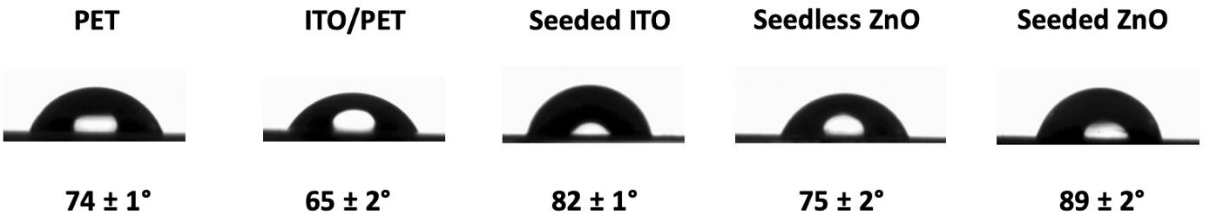

Figure S6. Images of the sessile droplets on the different types of surfaces used in this study.

5. EDX characterization

Table S2. EDX elemental analysis of NCs in ZnO seedless and ZnO seeded samples.

Atomic Percentage Values (at. %)	seedless ZnO	seeded ZnO
**C**	63.14	52.55
**O**	30.35	35.36
**Mn**	0.00	0.05
**Zn**	1.20	2.71
**In**	4.93	8.63
**Sn**	0.38	0.70

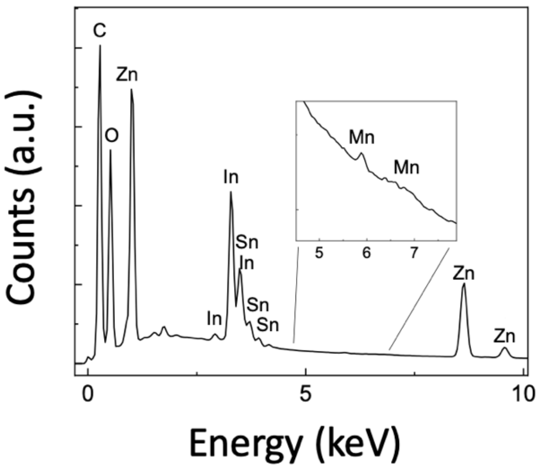

Figure S7. EDX characterization of the NCs observed on the seeded ZnO.

6. Electrical characterization

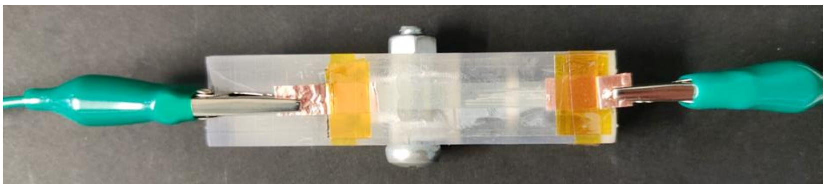

Figure S8. Optical picture of the sensor mounted on the 3D printed bending device.

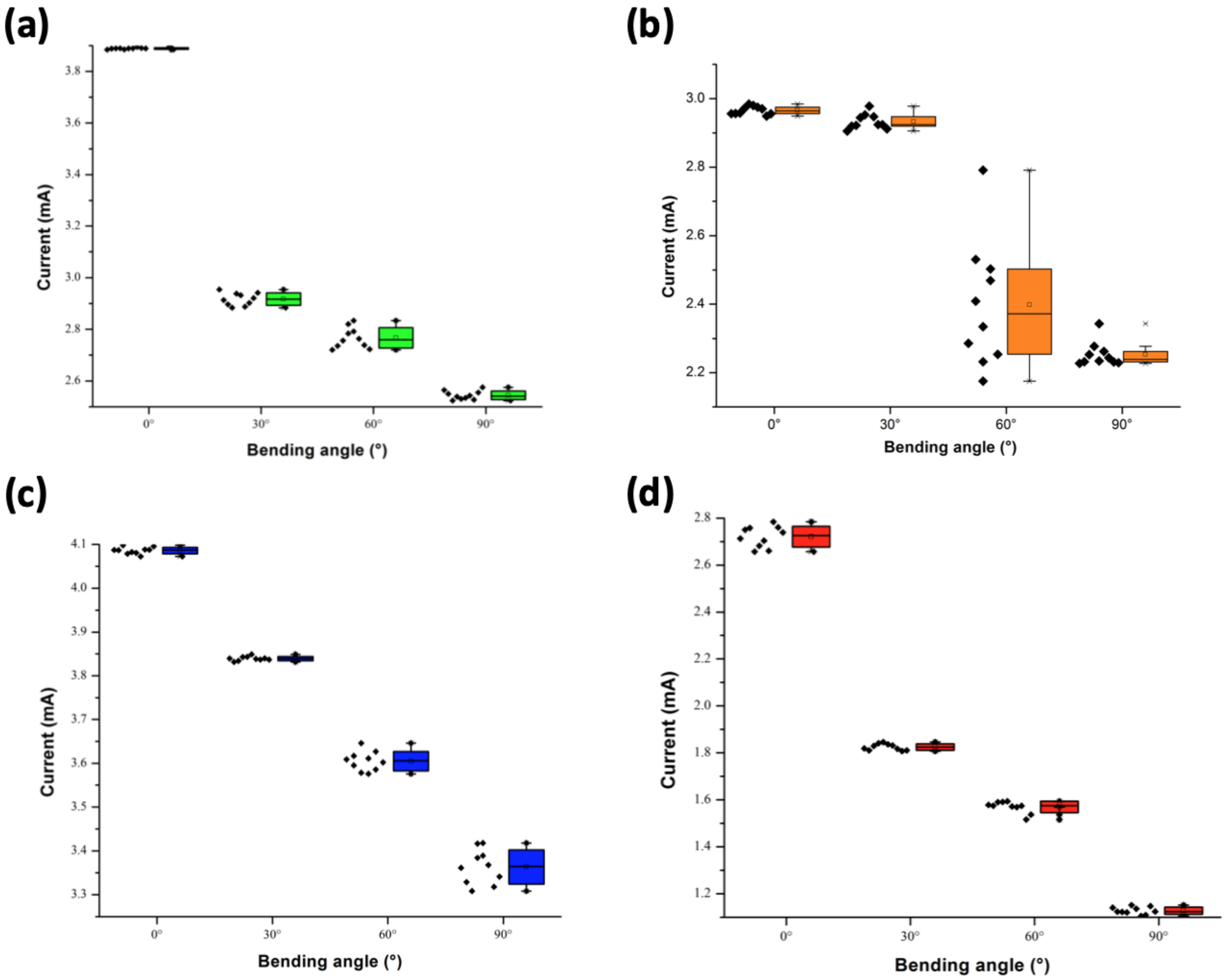

Figure S9. Statistical box plots showing ten measured current values along with their average at +2 V voltage bias for **(a)** ITO, **(b)** seeded ITO, **(c)** seedless ZnO and **(d)** seeded ZnO.

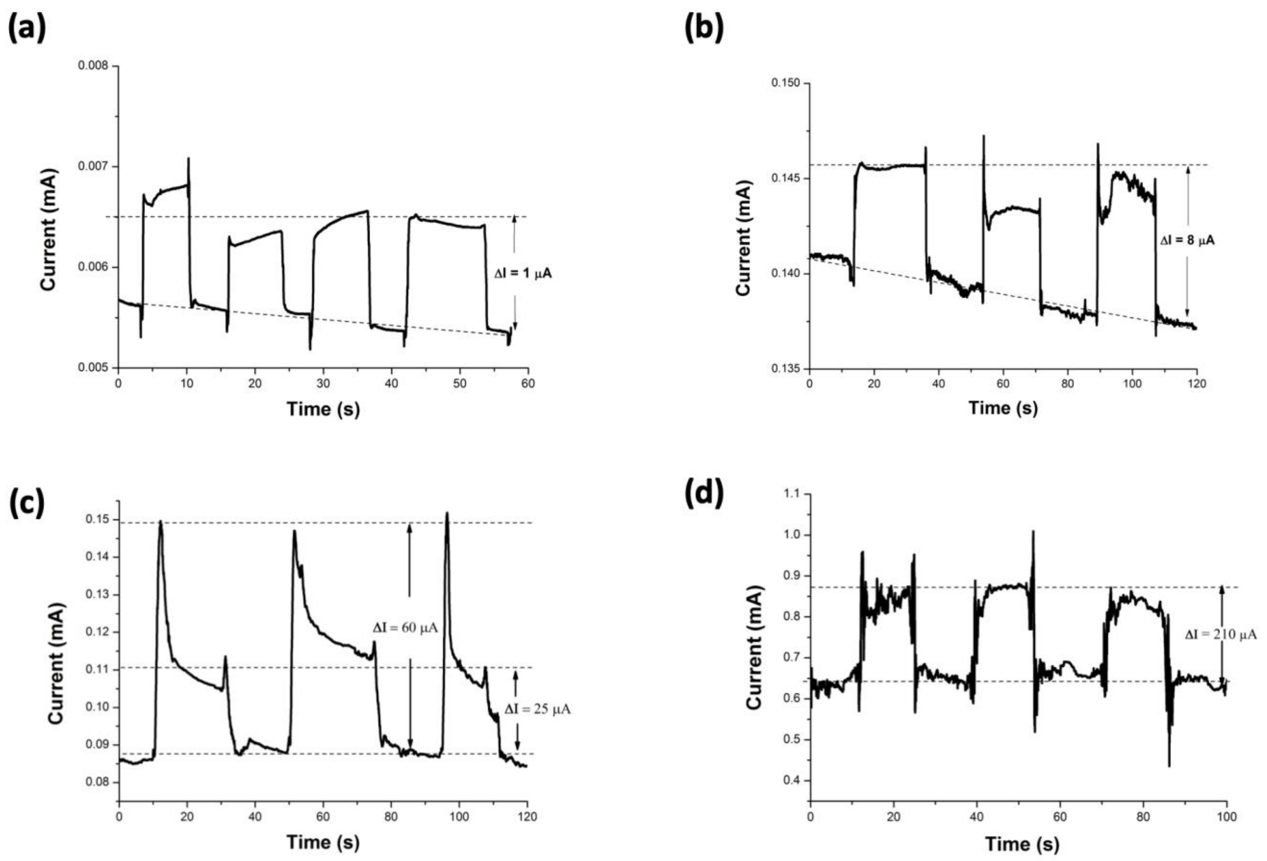

Figure S10. Chronoamperometric characterizations of **(a)** ITO and **(b)** seeded ITO, **(c)** seedless ZnO and **(d)** seeded ZnO devices allow evaluating consistent electrical responses.

7. Photocatalytic investigation

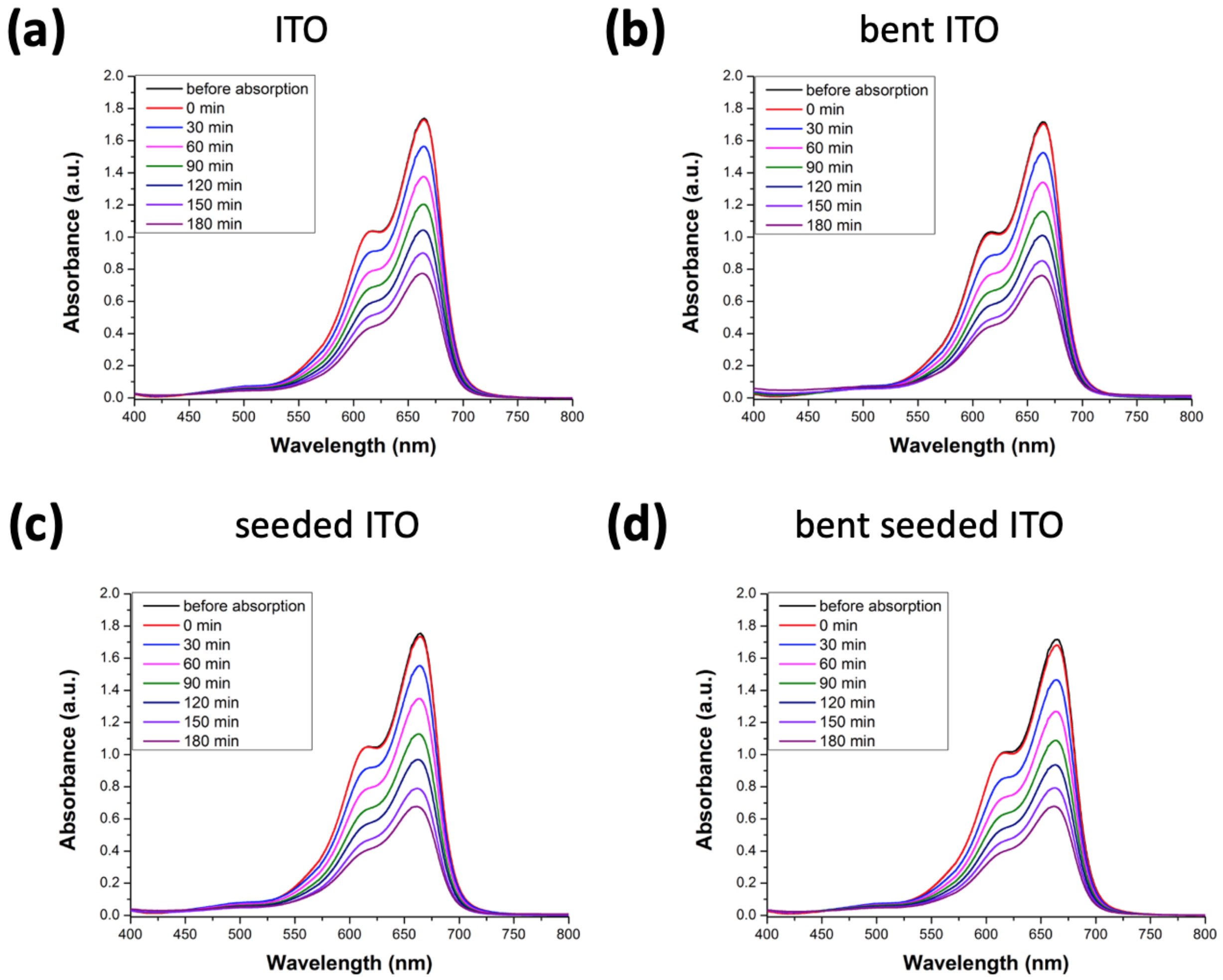

Figure S11. UV-vis spectra of MB (25 μM) solution were collected during the photodegradation process for **(a)** ITO, **(b)** bent ITO, **(c)** seeded ITO and **(d)** bent seeded ITO.

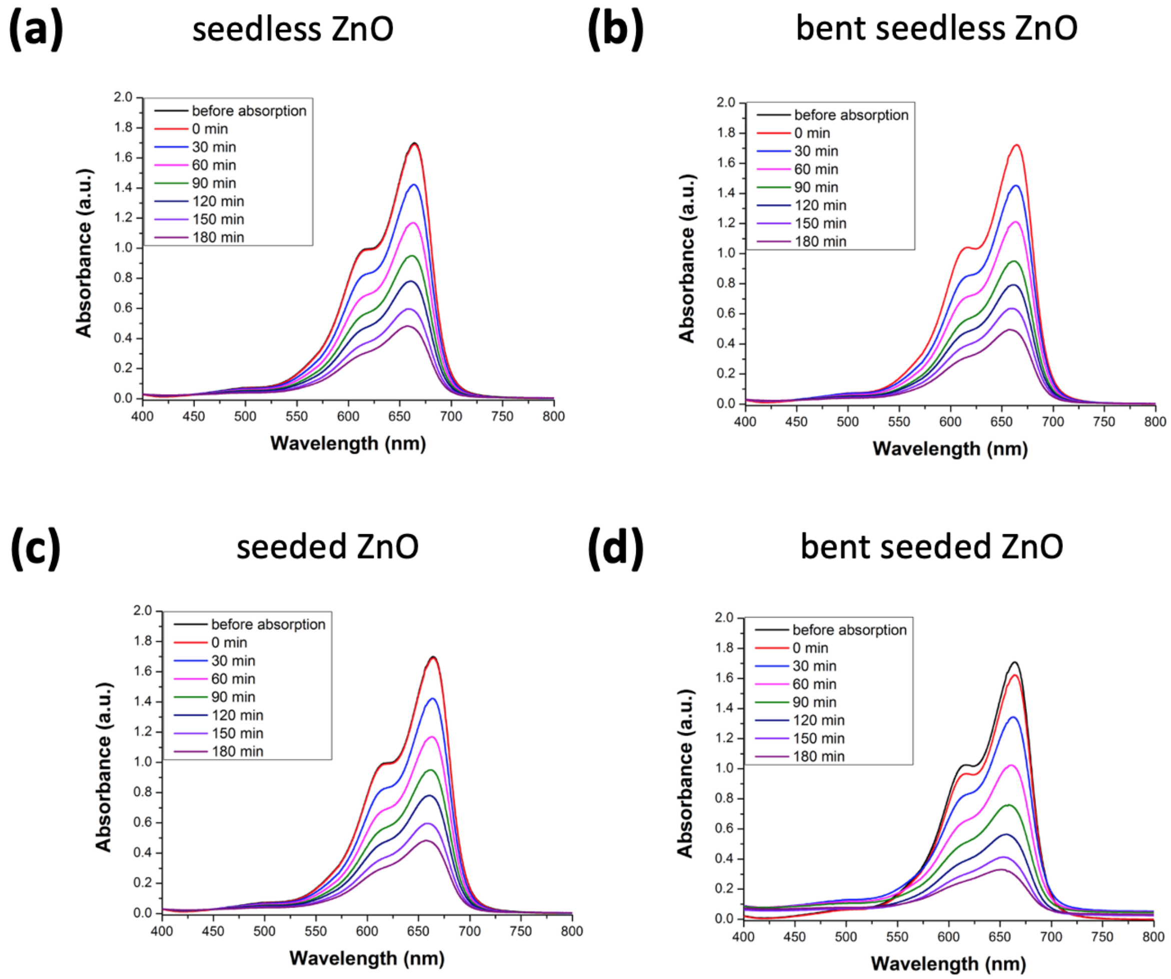

Figure S12. UV-vis spectra of MB (25 μM) solution were collected during the photodegradation process for **(a)** seedless ZnO, **(b)** bent seedless ZnO, **(c)** seeded ZnO and **(d)** bent seeded ZnO.

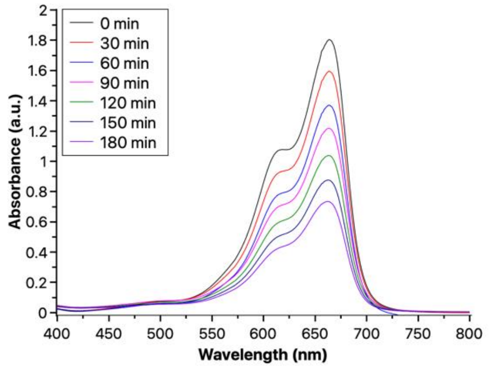

Figure S13. UV-vis spectra of MB (25 μM) solution were collected during the photodegradation process in the absence of ITO or ZnO/ITO.

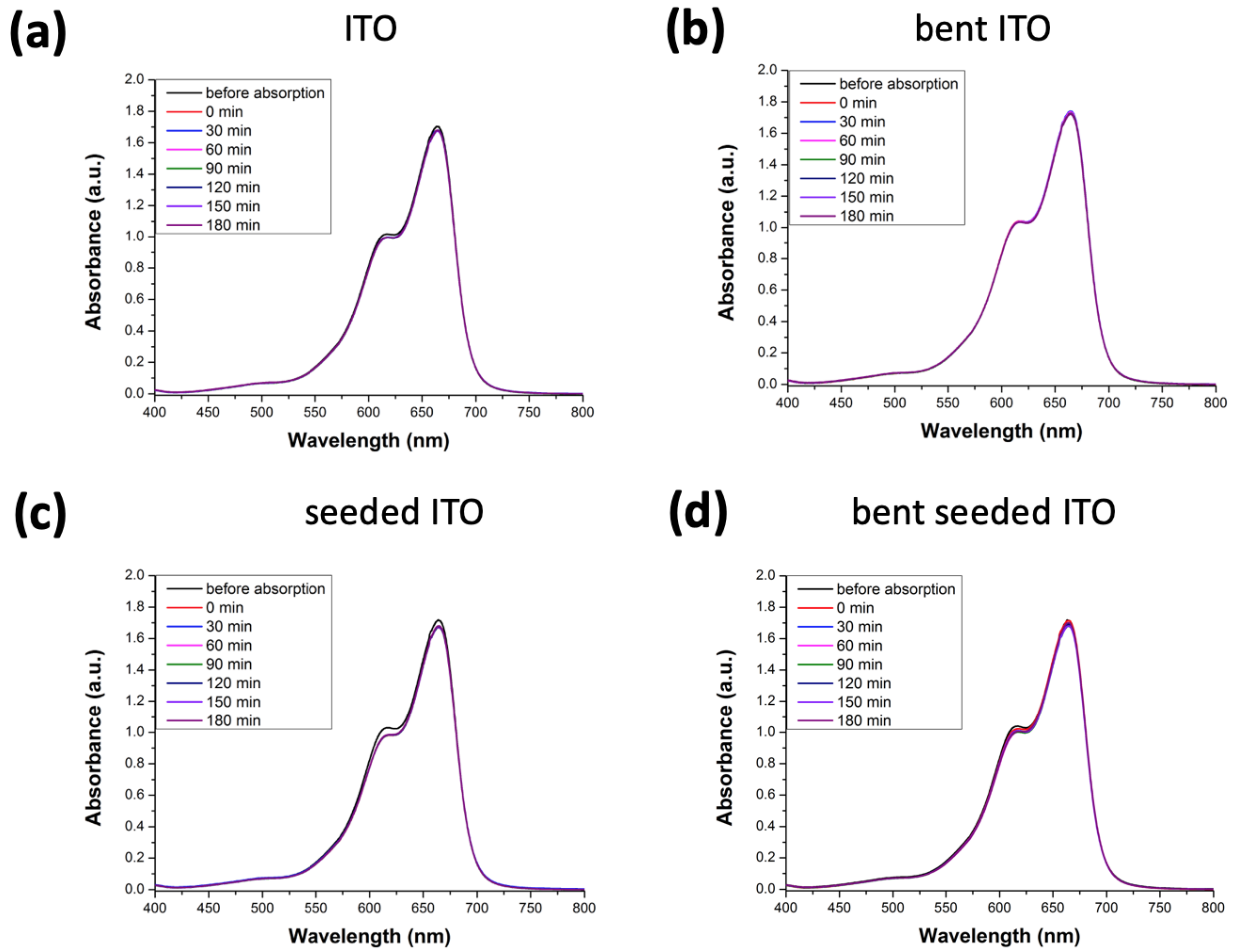

Figure S14. UV-vis spectra of MB (25 μM) solution were collected under dark conditions for **(a)** ITO, **(b)** bent ITO, **(c)** seeded ITO and **(d)** bent seeded ITO.

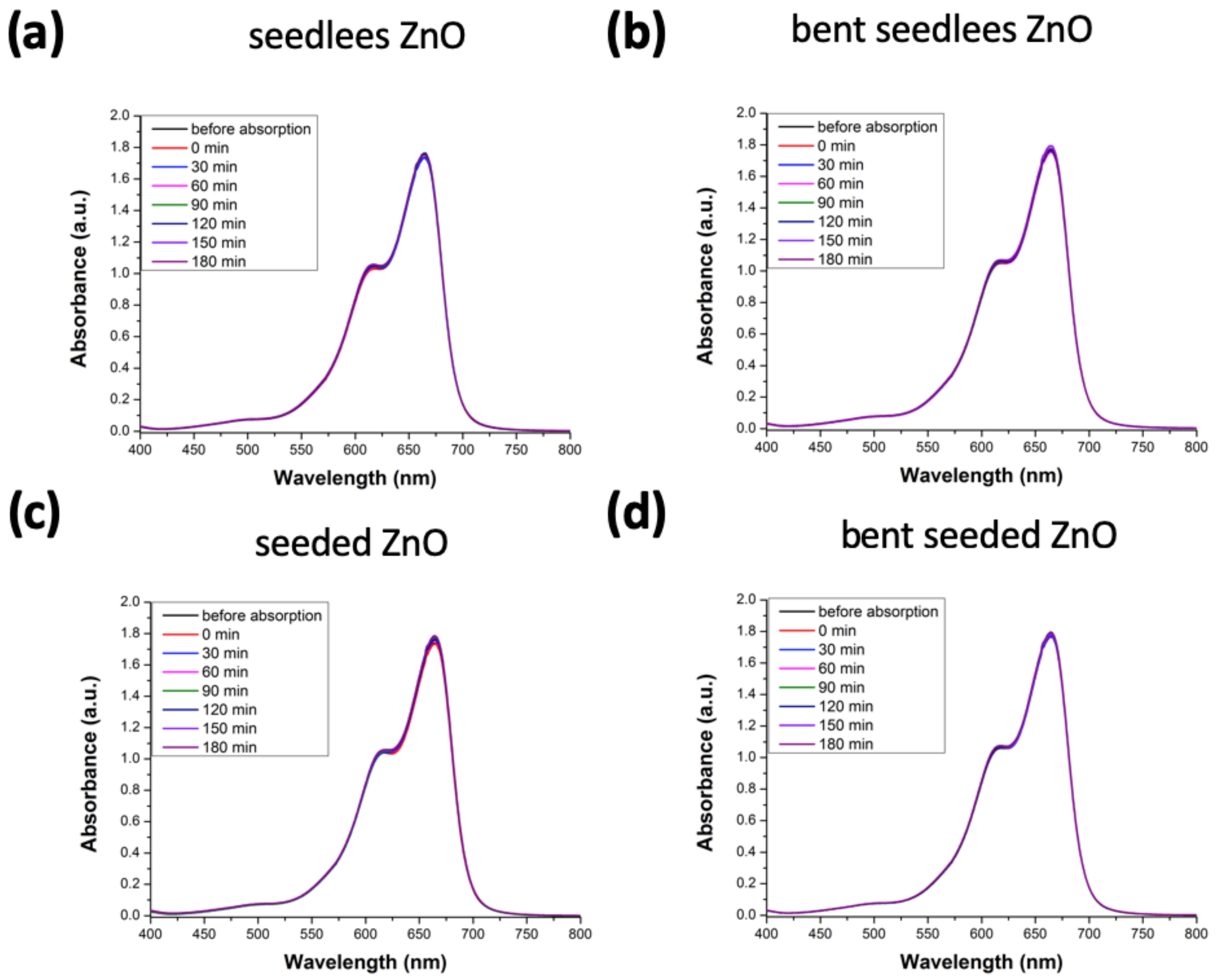

Figure S15. UV-vis spectra of MB (25 µM) solution were collected under dark conditions for **(a)** seedless ZnO, **(b)** bent seedless ZnO, **(c)** seeded ZnO and **(d)** bent seeded ZnO.

Table S3. Summary of ZnO catalyzed dyes photodegradation efficiencies in the literature. The reports are categorized according to the photocatalyst/dye couple, light source, dark absorption time (min), degradation time (min), percentage degradation, apparent rate constant (min^{-1}).

**Reference**	**Photocatalyst/dye**	**Light source**	**Dark absorption time (min or hours)**	**Degradation time (min or hours)**	**% Degradation**	**Apparent rate constant (min^{-1})**
M. Nasr-Esfahani et al. [10]	ZnO-ITO/Direct Sky Blue 5B	200 W high-pressure mercury lamp	n.r.	50 min	40%	0.0301
C.J. Chang et al. [11]	Ag NPs-ZnO-ITO/Methyl Orange (MO)	UV light (365 nm, 14.3 mW/cm^2)	n.r.	200 min	~100%	n.r.
R. Cui et al. [12]	ZnO NWs-AZO-ITO)/Rhodamine B	500 W Xenon lamp range at 190–1100 nm, 425 mW/cm^2	30 min	180 min	~100%	0.015
G. Wang [13]	ZnO NWs on Si(100) or (ITO)/MO	50 W Xe light equipped with a UV cut-off-filter (λ > 400 nm)	60 min	120 min	60%	0.0038
L. Fan [14]	Mn-doped ZnO nanorods on ITO/MB (Methylene blue)	UV light	n.r.	60 min	38% (undoped) 82% (Mn-doped)	0.0942 (undoped) 0.3114 (Mn-doped)
Q. Ma et al.[15]	Mn-doped ZnO nanoflowers/MB	a 250 W high pressure mercury lamp with a 420 nm cutoff	n.r.	240 min	90% (Mn-doped)	n.r.
M.K. Choudhary et al.[16]	Au-ZnO/MB	200 W tungsten filament lamp (Philips)	n.r.	180 min	40% (pure ZnO) 76% (Au-ZnO)	0.0036 (pure ZnO) 0.0115 (Au-ZnO)
M. Segovia [17]	Cu_2O- ZnO/MB	150W ozone-free xenon bulb, intensity of 1000 W/m^2	120 min	300 min	30% (pure ZnO) 80% (Cu_2O -ZnO)	0.0012 (pure ZnO) 0.0046 (Cu_2O -ZnO)
T. Wang et al.[18]	ZnO-ITO/ MB	350 W xenon lamp as the light source	30 min	3.5 hours	100%	n.r.
X. Wang et al. [19]	ZnO-ITO/ MB	xenon lamp	30 min	240 min	90.3%	n.r.

O.E. Cigarroa-Mayorga et al. [20]	ZnO NWs decorated with Fe_2O_3 NPs/MB	light source of 254 nm (UV-lamp) and 15 W of power.	30 min	180 min	36% (bare)<br>90% (doped)	0.024 (bare)<br>0.0133 (doped)
F.Z. Nouasria et al. [21]	Cu doped ZnO NPs/MB	four UV lamps (365 nm) with a total power of 30 W or sunlight	n.r.	150 min	UV light<br>74%<br>(pure ZnO)<br>95% (Cu-doped ZnO)<br><br>Sunlight<br>53%<br>(pure ZnO)<br>86% (Cu-doped ZnO)	UV light<br>0.0056 (pure ZnO)<br>0.0091 (Cu-doped ZnO)<br><br>Sunlight<br>0.0057 (Cu-doped ZnO)
P. Rong et al.[22]	Cu–ZnO/MB	500 W Xe-lamp	30 min	7 hours	45.6% (MB only)<br>69.1% (ZnO/graphene coated PET)<br>83.6% (Cu-ZnO/ graphene coated PET)	n.r.
K.K. Supin al.[23]	ZnO NPs/MB	Sunlight	n.r.	90 min	96-98%	n.r.
L. Motelica et al.[24]	ZnO NPS/MB	Visible light fluorescent lamp of 160 W	30 min	60 min	99%	0.0077
This work	ZnO ITO/MB	Solar simulator (150 W Xenon arc lamp and an AM 1.5 G filter)	60 min	180 min	72% (Seedless ZnO)<br>75% (Seeded ZnO)	0.0072 (Seedless ZnO) and 0.0077 (Seeded ZnO)

References

(1) Bazargan, A. M.; Sharif, F.; Mazinani, S.; Naderi, N. A High Quality ITO/PET Electrode for Flexible and Transparent Optoelectronic Devices. *J. Mater. Sci. Mater. Electron.* **2017**, *28* (3), 2962–2969. https://doi.org/10.1007/s10854-016-5881-7.

(2) Zhang, X. L.; Qiao, R.; Li, V.; Qiu, R.; Kang, Y. S. Synthesis and Characterization of Nickel-Doped ZnO Nanocrystals. *Mater. Res. Soc. Symp. Proc.* **2007**, *957*, 353–358. https://doi.org/10.4236/wjcmp.2012.24035.

(3) Dos Santos Pereira, A. P.; Da Silva, M. H. P.; Lima, É. P.; Dos Santos Paula, A.; Tommasini, F. J. Processing and Characterization of PET Composites Reinforced with Geopolymer Concrete Waste. *Mater. Res.* **2017**, *20*, 411–420. https://doi.org/10.1590/1980-5373-MR-2017-0734.

(4) Wu, Y.; Kong, L.; Zhang, X.; Guo, Y.; Sun, Y.; Zhao, C.; Chen, W.; Zuo, Y.; Li, C. Mesoporous Mn-Based Multi-Component Metal Oxide for Fast Chemical Warfare Agent Degradation. *AIP Adv.* **2022**, *12* (3). https://doi.org/10.1063/5.0083018.

(5) Ananth, M. V.; Pethkar, S.; Dakshinamurthi, K. Distortion of MnO6 Octahedra and Electrochemical Activity of Nstutite-Based MnO_2 Polymorphs for Alkaline Electrolytes - An FTIR Study. *J. Power Sources* **1998**, *75* (2), 278–282. https://doi.org/10.1016/S0378-7753(98)00100-1.

(6) Li, D.; Neumann, A. W. Contact Angles on Hydrophobic Solid Surfaces and Their Interpretation. *J. Colloid Interface Sci.* **1992**, *148* (1), 190–200. https://doi.org/https://doi.org/10.1016/0021-9797(92)90127-8.

(7) Hennig, A.; Eichhorn, K. J.; Staudinger, U.; Sahre, K.; Rogalli, M.; Stamm, M.; Neumann, A. W.; Grundke, K. Contact Angle Hysteresis: Study by Dynamic Cycling Contact Angle Measurements and Variable Angle Spectroscopic Ellipsometry on Polyimide. *Langmuir* **2004**, *20* (16), 6685–6691. https://doi.org/10.1021/la036411l.

(8) Kim, J. S.; Friend, R. H.; Cacialli, F. Surface Energy and Polarity of Treated Indium–Tin–

Oxide Anodes for Polymer Light-Emitting Diodes Studied by Contact-Angle Measurements. *J. Appl. Phys.* **1999**, *86* (5), 2774–2778. https://doi.org/10.1063/1.371124.

(9) Papakonstantinou, D.; Amanatides, E.; Mataras, D.; Ioannidis, V.; Nikolopoulos, P. Improved Surface Energy Analysis for Plasma Treated PET Films. *Plasma Process. Polym.* **2007**, *4* (S1), S1057–S1062. https://doi.org/https://doi.org/10.1002/ppap.200732405.

(10) Nasr-Esfahani, M.; Nourmohammadi, A. ZnO/ITO Interface Nanostructure Films for the Photocatalytic Degradation of a Textile Dye. *React. Kinet. Mech. Catal.* **2012**, *107* (1), 79–88. https://doi.org/10.1007/s11144-012-0450-4.

(11) Chang, C. J.; Hsu, M. H.; Weng, Y. C.; Tsay, C. Y.; Lin, C. K. Hierarchical ZnO Nanorod-Array Films with Enhanced Photocatalytic Performance. *Thin Solid Films* **2013**, *528*, 167–174. https://doi.org/10.1016/j.tsf.2012.09.083.

(12) Cui, R.; Shen, K.; Xu, M.; Xiang, D.; Xu, Q. Enhancing Photocatalytic Activity of ZnO Nanowires by Embedding ITO Layer as a Photogenerated Electron Collecting Layer. *Mater. Sci. Semicond. Process.* **2016**, *43*, 155–162. https://doi.org/10.1016/j.mssp.2015.12.003.

(13) Wang, G.; Li, Z.; Li, M.; Feng, Y.; Li, W.; Lv, S.; Liao, J. Synthesizing Vertical Porous ZnO Nanowires Arrays on Si/ITO Substrate for Enhanced Photocatalysis. *Ceram. Int.* **2018**, *44* (2), 1291–1295. https://doi.org/10.1016/j.ceramint.2017.08.035.

(14) Fan, L.; Wang, J.; Qiu, N.; Liu, Y.; Zhang, X. Photocatalytic Activities of Vertically Aligned Manganese-Doped ZnO Nanorods Synthesized on ITO Film by Electrochemical Technique. *Int. J. Electrochem. Sci.* **2019**, *14*, 10862–10862. https://doi.org/10.20964/2019.12.09.

(15) Ma, Q.; Lv, X.; Wang, Y.; Chen, J. Optical and Photocatalytic Properties of Mn Doped Fl Ower-like ZnO Hierarchical Structures. *Opt. Mater. (Amst).* **2016**, *60*, 86–93. https://doi.org/10.1016/j.optmat.2016.07.014.

(16) Choudhary, M. K.; Kataria, J.; Sharma, S. Novel Green Biomimetic Approach for Preparation of Highly Stable Au-ZnO Heterojunctions with Enhanced Photocatalytic Activity. *ACS Appl. Nano Mater.* **2018**, *1* (4), 1870–1878.

https://doi.org/10.1021/acsanm.8b00272.

(17) Segovia, M.; Alegría, M.; Aliaga, J.; Celedon, S.; Ballesteros, L.; Sotomayor-Torres, C.; González, G.; Benavente, E. Heterostructured 2D ZnO Hybrid Nanocomposites Sensitized with Cubic Cu_2O Nanoparticles for Sunlight Photocatalysis. *J. Mater. Sci.* **2019**, *54* (21), 13523–13536. https://doi.org/10.1007/s10853-019-03878-x.

(18) Wang, T.; Lu, Z.; Wang, X.; Zhang, Z.; Zhang, Q.; Yan, B.; Wang, Y. A Compound of ZnO/PDMS with Photocatalytic, Self-Cleaning and Antibacterial Properties Prepared via Two-Step Method. *Appl. Surf. Sci.* **2021**, *550*, 149286. https://doi.org/10.1016/j.apsusc.2021.149286.

(19) Wang, X.; Li, X.; Zhang, Q.; Lu, Z.; Song, H.; Wang, Y. Electrodeposition of ZnO Nanorods with Synergistic Photocatalytic and Self-Cleaning Effects. *J. Electron. Mater.* **2021**, *50* (8), 4954–4961. https://doi.org/10.1007/s11664-021-08958-w.

(20) Cigarroa-Mayorga, O. E. Enhancement of Photocatalytic Activity in ZnO NWs Array Due to Fe2O3 NPs Electrodeposited on the Nanowires Surface: The Role of ZnO-Fe_2O_3 Interface. *Mater. Today Commun.* **2022**, *33*, 104879. https://doi.org/10.1016/j.mtcomm.2022.104879.

(21) Nouasria, F. Z.; Selloum, D.; Henni, A.; Tingry, S.; Hrbac, J. Improvement of the Photocatalytic Performance of ZnO Thin Films in the UV and Sunlight Range by Cu Doping and Additional Coupling with Cu_2O. *Ceram. Int.* **2022**, *48* (9), 13283–13294. https://doi.org/10.1016/j.ceramint.2022.01.207.

(22) Rong, P.; Jiang, Y. F.; Wang, Q.; Gu, M.; Jiang, X. L.; Yu, Q. Photocatalytic Degradation of Methylene Blue (MB) with Cu1-ZnO Single Atom Catalysts on Graphene-Coated Flexible Substrates. *J. Mater. Chem. A* **2022**, *10* (11), 6231–6241. https://doi.org/10.1039/d1ta09954j.

(23) Supin, K. K.; Parvathy Namboothiri, P. N.; Vasundhara, M. Enhanced Photocatalytic Activity in ZnO Nanoparticles Developed Using Novel Lepidagathis Ananthapuramensis Leaf Extract. *RSC Adv.* **2023**, *13* (3), 1497–1515. https://doi.org/10.1039/d2ra06967a.

(24) Motelica, L.; Oprea, O.-C.; Vasile, B.-S.; Ficai, A.; Ficai, D.; Andronescu, E.; Holban, A. M.

Antibacterial Activity of Solvothermal Obtained ZnO Nanoparticles with Different Morphology and Photocatalytic Activity against a Dye Mixture: Methylene Blue, Rhodamine B and Methyl Orange. *Int. J. Mol. Sci.* **2023**, *24* (6), 5677. https://doi.org/10.3390/ijms24065677.
